# HIV Infection and Antiretroviral Therapy Impair Liver Function in People Living with HIV: Systematic Review and Meta-Analysis

**DOI:** 10.3390/ph18070955

**Published:** 2025-06-25

**Authors:** Kay-Lee E. Strauss, Wendy N. Phoswa, Sidney Hanser, Kabelo Mokgalaboni

**Affiliations:** 1 Department of Life and Consumer Sciences, College of Agriculture and Environmental Sciences, University of South Africa, Florida Campus, Roodepoort 1710, South Africa; 68188242@mylife.unisa.ac.za (K.-L.E.S.);; 2 Department of Physiology and Environmental Health, University of Limpopo, Sovenga 0727, South Africa

**Keywords:** antiretroviral therapy, AST, ALT, ALP, hepatotoxicity, HIV, liver function, systematic review, meta-analysis

## Abstract

**Background:** The use of antiretroviral therapy (ART) has improved the lives of people living with HIV (PLWH). However, its use is associated with secondary complications, notably hepatotoxicity. This systematic review and meta-analysis assess the effects of HIV infection and ART on liver function in PLWH. **Method:** A comprehensive literature search was performed in PubMed, Scopus, and Google Scholar from inception to 12 February 2025. Studies analyzing liver enzymes such as aspartate aminotransferase (AST), alanine aminotransferase (ALT), and alkaline phosphatase (ALP) in PLWH undergoing ART, those who are ART-naïve, and HIV-negative individuals were considered. Data analysis was performed using a meta-analysis web tool, and the results were reported as standardized mean differences (SMDs) and 95% confidence intervals (CIs). **Results:** Twenty-six studies were included in the meta-analysis. The findings showed an increase in AST, SMD = 1.85 (0.93 to 2.78, *p* < 0.0001, I^2^ = 93.8%), and ALT, SMD = 2.65 (1.25 to 4.04, *p* = 0.0002, I^2^ = 97.8%) in PLWH who were naïve compared with those who were HIV negative. Additionally, there was a pronounced elevation in AST, SMD = 1.49 (0.48 to 2.50, *p* = 0.0038, I^2^ = 98%); ALT, SMD = 2.30 (1.14 to 3.45, *p* < 0.0001, I^2^ = 98%); and ALP, SMD = 1.40 (0.55 to 2.26, *p* < 0.01, I^2^ = 97%) in PLWH exposed to ART compared with HIV-negative individuals. However, there was no significant difference in ALP, SMD = 0.53 (–0.92 to 1.98, *p* = 0.4726, I^2^ = 98%) between PLWH who were ART-naïve and HIV-negative individuals. **Conclusions:** The results show that HIV infection and ART administration are associated with elevated liver function test enzymes, suggesting that each may contribute to liver dysfunction among PLWH. These results highlight the dual risk posed by HIV infection and ART exposure.

## 1. Introduction

The human immunodeficiency virus (HIV) targets human CD4 cells, thereby impairing the immune system and thus making individuals susceptible to infections [1,2,3]. If left untreated, HIV can progress into the advanced stage, acquired immune deficiency syndrome (AIDS) [2]. Interestingly, the introduction of antiretroviral therapy (ART) has improved the quality of life among people living with HIV (PLWH) [4]. ART is a group of HIV medications that improves the immune system and reduces morbidity and mortality by suppressing HIV replication [4]. However, many ART regimens are associated with adverse events, including hepatotoxicity and virologic failure, which can lead to liver injury or dysfunction [5,6]. This, in turn, raises concerns about the impact of prolonged exposure to ART on the overall health of the liver in PLWH.

Existing evidence has highlighted the hepatotoxic effects of a specific ART. For instance, a previous study has shown that the use of nevirapine-based therapy is associated with severe liver abnormalities [7]. It is worth noting that while newer ART regimes such as dolutegravir (DTG) have proven to reduce viral levels and stabilize CD4 count effectively, their overall effect on liver function in PLWH is concerning, especially if there is a co-infection with the hepatitis virus [8,9,10]. Among those with co-infection, the liver-related mortality rate is increasing compared with HIV-monoinfected individuals [11]. Other researchers suggest that the duration of ART exposure is a key factor, with prolonged exposure contributing to liver damage and dysfunction [12,13,14].

The liver is a vital organ in the metabolism and excretion of drugs, making it susceptible to the effects of medications, including ART [15]. This can result in liver dysfunction, subsequently leading to secondary complications due to impaired hepatic clearance and metabolic function [16]. Therefore, understanding the relationship between ART use and liver function is crucial for maximizing the treatment of HIV infection and reducing the likelihood of secondary complications. Liver function is evaluated by using biochemical markers, such as alanine aminotransferase (ALT), aspartate aminotransferase (AST), and alkaline phosphatase (ALP), and their elevation indicates hepatic impairment [17,18]. Previous evidence has reported abnormal liver enzymes in PLWH on highly active antiretroviral therapy (HAART) regimens, even in the absence of hepatitis C virus (HCV) or hepatitis B virus (HBV) co-infections [19]. HAART consists of a combination of two nucleoside reverse transcriptase inhibitors (NRTIs) and one drug from another class, such as non-nucleoside reverse transcriptase inhibitors (NNRTIs), nucleotide reverse transcriptase inhibitors (NtRTI), protease inhibitors (PIs), or integrase-nucleoside strand transfer inhibitors (INSTIs) [20,21]. All these ART regimens have different effects on the overall hepatic health [22,23]. For instance, NNRTIs, such as nevirapine, are associated with hepatic toxicity [24,25]. On the other hand, long-term exposure to NNRTIs is not associated with the risk of hepatotoxicity [26]. This provides an unclear picture of the overall effect of these NNRTIs on hepatic health, particularly in PLWH. NRTIs, such as zidovudine and didanosine, inhibit the enzyme mitochondrial polymerase gamma, thus impairing DNA synthesis [27,28]. This activity results in the accumulation of toxic products that present as hepatic steatosis, lactic acidosis, and liver failure [29]. Surprisingly, NRTI taken as pre-exposure prophylaxis (PrEP) even in healthy individuals presents with mitochondrial toxicity [30]. This suggests that NRTI could increase the risk of mitochondrial oxidative stress and hepatic health in those living with HIV.

Notably, while liver enzyme abnormalities are frequently observed in PLWH on ART, other studies have reported increased liver enzymes in PLWH who are ART-naïve [5,31]. Existing evidence has demonstrated a slight increase in liver enzyme abnormality in HAART-naïve individuals compared with PLWH on HAART regimens [5]. This suggests that hepatic dysfunction in PLWH may be attributed to factors other than ART exposure. In 2016, Shiferaw and colleagues reported an association of liver enzyme elevation, mainly AST and ALT, with CD4 count, male sex, opportunistic infections, and viral hepatitis [5]. Although some studies suggest that ART exerts a hepatoprotective effect [32,33], this claim remains inconclusive due to conflicting evidence. Other studies found no effect of HIV or ART on liver function [34,35], while others reported an increase in liver enzymes, suggesting an undesirable effect on hepatic health [36,37,38]. On the other hand, other researchers reported a reduced activity of ALP, AST, and ALT during the early and late stages of ART treatment [32,33]. This complex relationship makes it difficult to distinguish between liver dysfunction induced by HIV infection and that induced by ART exposure in PLWH. Given these inconsistencies, it is important to evaluate the overall effect of HIV and ART on liver function among PLWH by focusing on the main liver enzymes such as AST, ALT, and ALP.

- Aim and Objectives

This study aimed to evaluate the overall effect of HIV and ART on liver function among PLWH.

- Objectives

To determine AST, ALT, and ALP levels in HIV individuals on ART and those who are ART-naïve compared with HIV-negative individuals.

## 2. Materials and Methods

### 2.1. Information Sources, Search Strategy

This study adhered to the Preferred Reporting Items for Systematic Reviews and Meta-Analyses (PRISMA) guideline [39] (Supplementary File S1). The protocol was registered through the Open Science Framework (OSF) (https://doi.org/10.17605/OSF.IO/B8JHF) to ensure transparency. A thorough literature search was conducted independently by K.E.S. and K.M. on PubMed and Scopus utilizing Medical Subject Headings (MeSH) terms. The MeSH terms employed for the search included “HIV”, “Human Immunodeficiency Virus”, “ART”, “Antiretroviral Therapy”, and “Liver Function Test.” In addition, the relevant Boolean operators “OR” and “AND” were used to build the search. Additionally, Google Scholar was also used to search for relevant studies.

### 2.2. Eligibility Criteria and Selection Process

Studies were considered relevant and included if they satisfied the following PICOS criteria. The study population (P) consisted of PLWH (adolescents and adults); intervention groups (I) were either HAART/ART or ART-naïve; the control group (C) was HIV negative; and the outcome of interest (O) was liver function, measured using surrogate markers such as AST, ALT, and ALP. The study design (S) included cross-sectional, case control and cohorts. Studies were excluded if they were conducted on HIV patients without ART exposure, without an HIV negative group as a control, studies in animals, or those not reporting any marker of liver function. This process was undertaken by two independent researchers (K.-L.E.S. and K.M.) by screening the title and abstract, followed by full text screening. To resolve any disagreement and ensure accuracy, a third independent researcher (W.N.P.) participated in the screening and selection process.

### 2.3. Data Items and Extraction

Two independent researchers (K.-L.E.S. and K.M.) used an Excel spreadsheet to extract data from individual studies. The two spreadsheets were compared, and if there was disagreement about variables and a study, a third independent researcher, W.N.P., was invited to assess the study and the variables in question before making a conclusion. The primary data items extracted from each study included the main author’s family name, country of publication, study design, population size, age of participants, gender (number of males and proportion) in the ART group, ART and duration of intervention, class of ART, CD4 counts, findings, mean, standard deviation (SD), and sample size of AST, ALT, and APL.

### 2.4. Quality Assessment and Risk of Bias

The methodological quality was assessed following the Newcastle–Ottawa Scale (NOS) [40]. The scale comprises four main domains, such as selection, comparability, and exposure. Within each domain, a couple of items were considered, and these were rated with stars. Studies that received 7–9 stars were regarded as high quality (low risk of bias), 4 to 6 stars were classified as moderate quality (moderate risk of bias), and those between 0 and 3 stars were considered low quality (high risk of bias).

### 2.5. Synthesis Method and Statistical Analysis

Meta-analysis was performed when more than two studies reported the same outcomes (AST, ALT, and ALP). An online meta-analysis web tool, freely accessible, was utilized for data analysis [41]. We determined the effect estimates for all liver enzymes by computing the mean, SD, and sample size from each group. Where median and range were reported in the original study instead of mean and SD, we estimated the mean and SD from the median and range following the guidelines established by Hozo et al., 2005 [42]. In instances where the original study provided the standard error of the mean (SEM) instead of SD, we calculated the SD using the formula SD = SEM × √n, where n is the sample size of the specific group (ART, ART-naïve, or HIV negative) [43]. In studies with several ART arms, we adopted the Cochrane method to combine the data sequentially into one treatment group (accessed 12 February 2025) (https://www.statstodo.com/CombineMeansSDs.php). For effect size, the standardized mean difference (SMD) was estimated using Hedges g or Cohen’s D approach based on the number of studies. When the number of studies was ≤20, Hedges g was used due to its reliability. For analysis with more than 20 studies, Cohen’s d was preferred. The SMD was then interpreted as follows: 0.2 and 0.5 were considered small and medium effects, respectively. Meanwhile, 0.8 and above were considered a large effect size. We utilized the I^2^ statistic test to evaluate statistical heterogeneity [44]. The I^2^ values of ≤50% and ≥75% were classed as low and substantial statistical heterogeneity, respectively. In the case of high heterogeneity, subgroup analysis was performed based on study design, continent of publication, sample size, gender distribution, and class of ART. Publication bias was evaluated graphically through funnel plots and statistically through Egger’s test, and an Egger *p*-value of less than 0.05 supported the presence of bias [45]. The trim and fill method was used to adjust for publication bias. Sensitivity analysis was also conducted to evaluate the stability of the effect size by removing one study at once and re-analyzing the overall effect. *p*-Values of less than 0.05 were deemed statistically significant.

## 3. Results

### 3.1. Literature Search and Study Selection

Our initial search across the PubMed and Scopus databases yielded 278 records. Additionally, we searched for studies on Google Scholar, and 26 relevant studies were identified. Initially, using an Excel sheet, we identified and removed 5 duplicate records from both databases. After screening titles, abstracts, and keywords, we found 2 records irrelevant to our research question.

**Figure 1:**
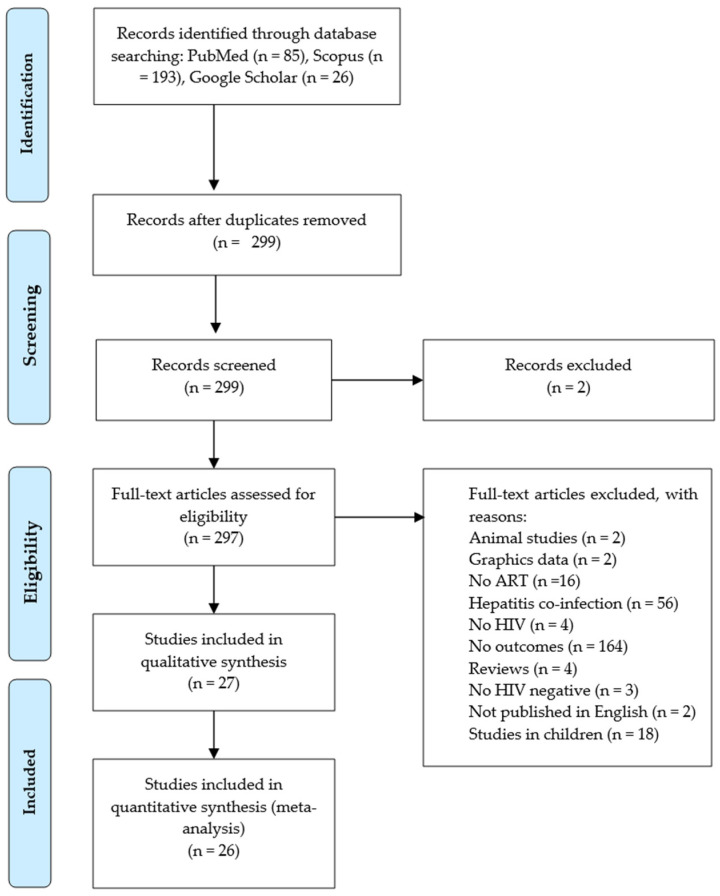
A PRISMA flow diagram showing the study selection procedure.

Consequently, 299 records remained and underwent independent screening by K.-L.E.S. and K.M. Of the latter records, 271 were excluded for various reasons, including studies in animal models, studies in children, graphical presentation of data, hepatitis co-infection, no ART regimens, no HIV diagnosis, no liver function tests as outcomes of interest, study not published in English, and review studies. Only 26 studies [32,34,35,36,37,38,46,47,48,49,50,51,52,53,54,55,56,57,58,59,60,61,62,63,64,65] were found to be relevant to this study (Figure 1).

### 3.2. The Demographic Features of the Studies

We examined data from 26 studies [32,34,35,36,37,38,46,47,48,49,50,51,52,53,54,55,56,57,58,59,60,61,62,63,64,65], published from 2007 to 2024, assessing the impact of ART on liver function in PLWH (Table S1). The sample sizes exhibited considerable variability across studies, ranging from small samples [62] to larger cohorts [38]. The total sample size comprised 4139 individuals, where all cohorts within the research comprised PLHIV on ART, ART-naïve people, and HIV-negative individuals. The research investigated the effects of ART on many demographic groups, including PLWH, ART-naïve individuals, HAART-naïve individuals, HIV-negative controls, pregnant women, and children affected by HIV. The published studies employed diverse research methodologies, such as case control [34,52,58,62,63] and cohorts [46,48,51], with most studies fulfilling the requirements of a cross-sectional design [32,34,35,36,37,38,47,49,50,53,54,55,56,57,59,60,61,64,65]. The evidence was scattered across six countries, with the majority carried out in Nigeria [32,34,37,38,47,48,49,51,54,55,57,60,61,62,64,65], succeeded by four in India [35,50,53,63], and one each in Brazil [46], Cameroon [36], Ghana [56], Kenya [59], and Libya [58]. The substantial quantity of Nigerian studies signifies a considerable emphasis on ART-related studies in the country. The age of the participants of the included studies ranged between 16 and 70 years, and the duration of ART treatment differed among studies, with some being short-term [48] and others being long-term [37].

### 3.3. Quality Assessment and Risk of Bias

The result of quality assessment and risk of bias among the included studies is presented in Supplementary File S2, Tables S2–S4. All cohort studies received 6 stars and were thus rated to be of moderate quality and risk of bias (File S2, Table S2). Cross-sectional studies varied in terms of quality and risk of bias, with 10 studies scoring 7 stars and thus classified as high quality and low risk of bias (File S2, Table S3), while case control studies received scores of 7 to 8 stars, reflecting high quality and low risk of bias (File S2, Table S4).

### 3.4. The Effect of HIV and ART on Aspartate Aminotransferase

**Figure 2:**
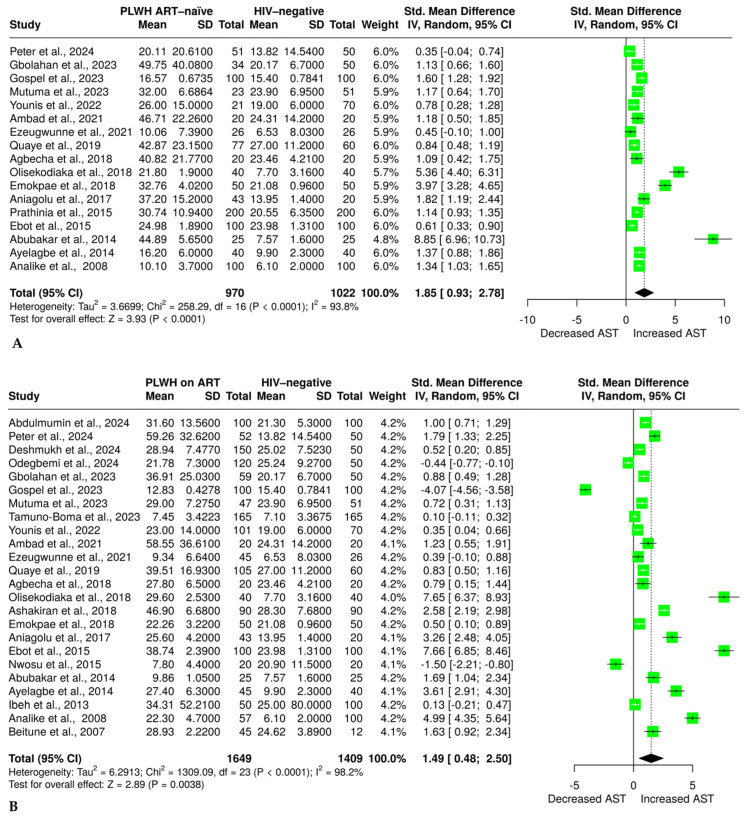
(**A**) Random effect meta-analysis evaluating the effect of HIV on aspartate aminotransferase in ART-naïve PLWH compared with the HIV-negative group [32,34,35,36,47,49,50,51,52,54,55,56,58,59,60,61,62]. (**B**) Random effect meta-analysis evaluating the effect of ART on aspartate aminotransferase in PLWH on ART compared with the HIV-negative group [32,34,35,36,37,38,46,47,48,49,51,52,53,54,55,56,58,59,60,61,62,63,64,65]. The solid line shows the line of no effect, the dashed line shows the effect size, the green block shows the weight of the study, the horizontal line crossing the green block shows the confidence intervals, and the diamond plot shows the combined effect size.

Seventeen studies [32,34,35,36,47,49,50,51,52,54,55,56,58,59,60,61,62] with 970 PLWH who were ART-naïve and 1022 HIV-negative individuals reported sufficient data on AST. The results from a random effects model indicate a significant increase in AST in ART-naïve PLWH compared with HIV-negative individuals, with an SMD of 1.85, 95% CI (0.93 to 2.78, *p* < 0.0001), as shown in Figure 2A. The evidence revealed significant heterogeneity, with an I^2^ of 93.8%. Additionally, to explore the effect of ART on AST, we found that 24 studies [32,34,35,36,37,38,46,47,48,49,51,52,53,54,55,56,58,59,60,61,62,63,64,65] had sufficient data on AST, of which 1649 PLWH were on ART and 1409 were HIV-negative individuals. The results revealed a significantly higher AST in the ART group compared with the HIV-negative group, SMD = 1.49, a 95% CI (0.48 to 2.50, *p* = 0.0038) (Figure 2B). The study reveals a significant level of heterogeneity (I^2^ = 98.2%).

Seventeen studies [32,34,35,36,47,49,50,51,52,54,55,56,57,58,59,60,61,62,63,64,65] with 970 PLWH who were ART-naïve and 1022 HIV-negative individuals reported sufficient data on AST. The results from a random effects model indicate a significant increase in AST in ART-naïve PLWH compared with HIV-negative individuals, with an SMD of 1.85, 95% CI (0.93 to 2.78, *p* < 0.0001), as shown in Figure 2A. The evidence revealed significant heterogeneity, with an I^2^ of 93.8%. Additionally, to explore the effect of ART on AST, we found that 24 studies [32,34,35,36,37,38,46,47,48,49,51,52,53,54,55,56,58,59,60,61,62,63,64,65] had sufficient data on AST, of which 1649 PLWH were on ART and 1409 were HIV-negative individuals. The results revealed a significantly higher AST in the ART group compared with the HIV-negative group, SMD = 1.49, a 95% CI (0.48 to 2.50, *p* = 0.0038) (Figure 2B). The study reveals a significant level of heterogeneity (I^2^ = 98.2%).

Seventeen studies [32,34,35,36,47,49,50,51,52,53,54,55,56,57,58,59,60,61,62,63,64,65] with 970 PLWH who were ART-naïve and 1022 HIV-negative individuals reported sufficient data on AST. The results from a random effects model indicate a significant increase in AST in ART-naïve PLWH compared with HIV-negative individuals, with an SMD of 1.85, 95% CI (0.93 to 2.78, *p* < 0.0001), as shown in Figure 2A. The evidence revealed significant heterogeneity, with an I^2^ of 93.8%. Additionally, to explore the effect of ART on AST, we found that 24 studies [32,34,35,36,37,38,46,47,48,49,51,52,53,54,55,56,58,59,60,61,62,63,64,65] had sufficient data on AST, of which 1649 PLWH were on ART and 1409 were HIV-negative individuals. The results revealed a significantly higher AST in the ART group compared with the HIV-negative group, SMD = 1.49, a 95% CI (0.48 to 2.50, *p* = 0.0038) (Figure 2B). The study reveals a significant level of heterogeneity (I^2^ = 98.2%).

### 3.5. Effect of HIV Infection and ART on ALT

**Figure 3:**
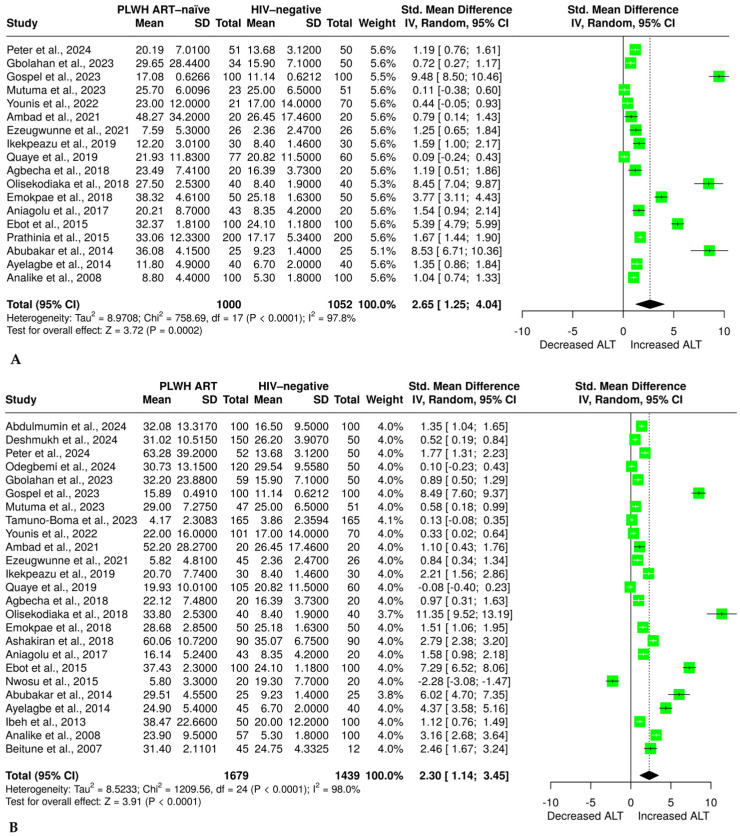
(**A**) Random effect meta-analysis evaluating the effect of no ART on alanine aminotransferase in ART-naïve PLWH compared with the HIV-negative group [32,34,35,36,47,49,50,51,52,54,55,56,57,58,59,60,61,62]. (**B**) Random effect meta-analysis evaluating the effect of ART on alanine aminotransferase in PLWH on ART compared with the HIV-negative group [32,34,35,36,37,38,46,47,48,49,51,52,53,54,55,56,57,58,59,60,61,62,63,64,65]. The solid line shows the line of no effect, the dashed line shows the effect size, the green block shows the weight of the study, the horizontal line crossing the green block shows the confidence intervals, and the diamond plot shows the combined effect size.

Evidence on the level of ALT from 18 studies [32,34,35,36,47,49,50,51,52,54,55,56,57,58,59,60,61,62] with 1000 PLWH who were ART-naïve and 1052 HIV-negative individuals was analyzed. The results showed a significant elevation in ALT in the ART-naïve group compared with the HIV-negative group (SMD = 2.65 (1.25 to 4.04, *p* = 0.0002)), as shown in Figure 3A. However, evidence showed a significant heterogeneity (I^2^ = 97.8%). We also found that 25 studies [32,34,35,36,37,38,46,47,48,49,51,52,53,54,55,56,57,58,59,60,61,62,63,64,65] had sufficient data on ALT, in 1679 PLWH on ART and 1439 HIV-negative individuals, and the data were subjected to random-effect model meta-analysis. The results revealed a significant increase in ALT levels in PLWH on ART compared with HIV-negative individuals (SMD = 2.30 (1.14 to 3.45, *p* < 0.0001)), as shown in Figure 3B. Consistently, the analyzed evidence revealed a significant heterogeneity (I^2^ = 98%).

### 3.6. The Effect of HIV Infection and ART on Alkaline Phosphatase

**Figure 4:**
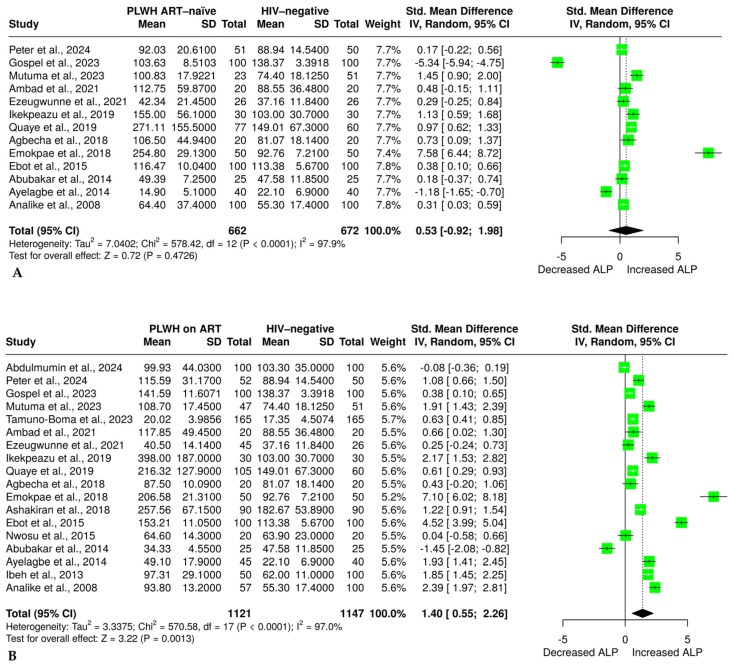
(**A**) Random effect meta-analysis evaluating the effect of no ART on alkaline phosphatase in ART-naïve PLWH compared with the HIV-negative group [32,34,35,36,47,49,52,55,56,57,59,60,62]. (**B**) Random effect meta-analysis evaluating the effect of ART on alkaline phosphatase in PLWH on ART compared with the HIV-negative group [32,34,35,36,37,38,47,48,49,52,53,55,56,57,59,60,62,65]. The solid line shows the line of no effect, the dashed line shows the effect size, the green block shows the weight of the study, the horizontal line crossing the green block shows the confidence intervals, and the diamond plot shows the combined effect size.

The effect of HIV infection on ALP was evaluated in 13 studies [32,34,35,36,47,49,52,55,56,57,59,60,62] comprising 662 ART-naïve PLWH and 672 HIV-negative individuals. The analysis of a random effects model revealed no significant difference in ALP levels between ART-naïve and HIV-negative individuals [SMD = 0.53 (–0.92 to 1.98, *p* = 0.4726)], as shown in Figure 4A. The evidence revealed a significant heterogeneity (I^2^ = 98%). Furthermore, the effect of ART on ALP was evaluated in 18 studies [32,34,35,36,37,38,47,48,49,52,53,55,56,57,59,60,62,65], with 1121 PLWH on ART and 1147 HIV-negative individuals. The results showed a statistically significant increase in ALP levels in PLWH on ART compared with HIV-negative individuals [SMD = 1.40 (0.55 to 2.26, *p* < 0.01)], as shown in Figure 4B. However, the accumulated evidence showed a significant level of heterogeneity (I^2^ = 97%).

### 3.7. Publication Bias and Sensitivity Analysis

**Figure 5:**
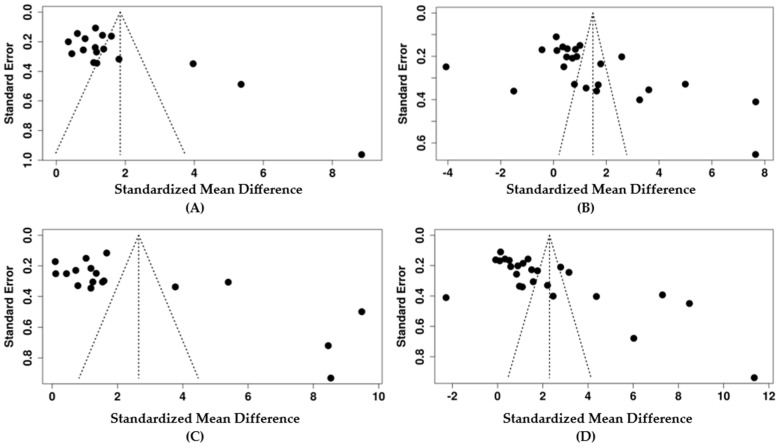

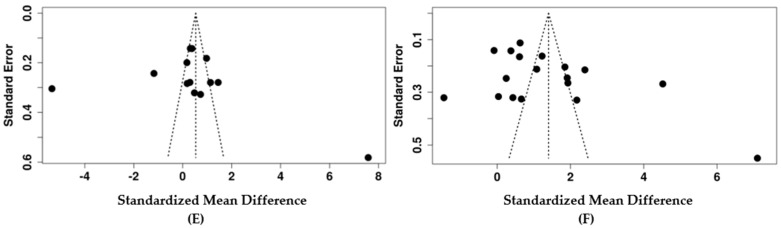
Funnel plots depicting the evidence of potential bias across the included studies in the meta-analysis. (**A**) Studies that assessed AST in ART-naïve PLWH compared with HIV-negative individuals. (**B**) Studies on the effect of PLWH on ART compared with HIV-negative individuals on AST. (**C**) Studies on ALT in ART-naïve PLWH compared with that in HIV-negative individuals. (**D**) Studies on the effect of ART on ALT in PLWH compared with that HIV-negative individuals. (**E**) Studies on ALP in ART-naïve PLWH compared with that in HIV-negative individuals. (**F**) Studies on the effect of ART on ALP. The individual dot shows the study included.

Publication bias was assessed through visual inspection of the funnel plot and Egger’s regression test. Briefly, when assessing the effect of HIV infection on AST, the funnel plot indicated a potential publication bias (Figure 5A). Egger’s test supported the presence of funnel plot asymmetry (intercept: 5.38, 95% CI: 1.45 to 9.31, *p* = 0.017). The result of the trim and fill method showed a change in effect size, SMD = 1.47, *p* < 0.05 (File S2, Figure S1A), while the effect was still large. The overall results suggest that there was moderate publication bias. For those PLWH on ART, the AST funnel plot showed potential publication bias (Figure 5B). Egger’s test supported the presence of funnel plot asymmetry (intercept: 9.46, 95% CI: 2.15 to 16.77, *p* = 0.019). However, the trim and fill method showed a decrease in effect size, SMD = 0.22, *p* > 0.05 (File S2, Figure S1C), compared with the initial SMD = 1.49. These discrepancies suggest that the initial effect size may have been increased due to unpublished studies. The sensitivity test revealed that the exclusion of a study by Gospel [60] was an outlier, and Nwosu [65], due to a small sample size, increased the effect size (1.73 and 1.62). In contrast, the exclusion of Ebot [36] as an outlier and Olisekodiaka [54] due to lower CD4 count in ART-naïve reduced the effect size (1.22 and 1.23, respectively) (File S2, Table S5). For studies on HIV infections on ALT, the funnel plot indicated a potential publication bias (Figure 5C). Egger’s test supported the presence of funnel plot asymmetry (intercept: 8.54, 95% CI: 2.23 to 14.86, *p* = 0.0017). However, the results of the trim and fill method did not alter the original effect size, suggesting that the effect size reflects the true effect, and therefore, the results are more reliable (File S2, Figure S1B). For the PLWH on ART on ALT, the funnel plot indicated a potential publication bias (Figure 5D). Egger’s test supported the presence of funnel plot asymmetry (intercept: 11.59, 95% CI: 6.43 to 16.75, *p* = 0). Additionally, a trim and ill test showed a decrease in effect size, SMD = 0.62, *p* > 0.05 (File S2 Figure S1D). This suggests that the initial effect size might have been attributed to unpublished studies. A sensitivity analysis also showed a minor change in effect size when some studies were excluded. However, the effect remained large, ranging from 1.93 to 2.48 (File S2, Table S6). Interestingly, no evidence of publication bias was observed on ALP from the evidence on HIV infection and those who were ART-naïve. The funnel plot did not indicate a potential publication bias (Figure 5E). Egger’s test did not support the presence of funnel plot asymmetry (intercept: 1.97, 95% CI: –9.59 to 13.53, *p* = 0.745). The funnel plot did not indicate a potential publication bias for studies on ART versus HIV negative (Figure 5F). This was supported by Egger’s test (intercept: 6.81, 95% CI: –0.11 to 13.74, *p* = 0.072).

### 3.8. Subgroup Analysis

A thorough subgroup analysis was performed to find the cause of observed statistical heterogeneity on AST, ALT, and ALP in PLWH who were exposed to ART, ART-naïve PLWH, and HIV-negative individuals. The analysis was based on study design, continent, sample size, and gender distribution, as presented in File S2, Figures S2–S7. Results on AST in HIV-ART-naïve when compared with HIV-negative results in the subgroup showed that case control (I^2^ = 31%) (File S2, Figure S2A), studies in Asian countries (I^2^ = 0%) (File S2, Figure S2B), those that did not state the gender (I^2^ was reduced from 94% to 63%) (File S2 Figure S2D), and studies that used NNRTI and NRTI from regimens were all factors contributing to heterogeneity (File S2 Figure S2E). On the other hand, no factors were found to be associated with heterogeneity on AST in PLWH on ART when compared with HIV negative (File S2, Figure S3A–E). This suggests an unexplained heterogeneity in AST in PLWH on ART. For ALT in the ART-naïve group, case control and Asian countries minimally contributed to the observed heterogeneity; post-subgroup I^2^ changed in the case control to 55% (File S2, Figure S4A) and 84% in Asian publications (File S2, Figure S4B). The studies that did not report the class of ART used were a source of heterogeneity (I^2^ = 0%) (File S1, Figure S4E). Additionally, those that used NNRTI and NRTI might have contributed minimally to the observed variation (I^2^ = 72.2%) (File S2 Figure S4E). For ALT in PLWH on ART, only case control studies showed minimal changes in heterogeneity from the original results (I^2^ = 86%) (File S2 Figure S5A). For ALP in ART-naïve, a case control changed I^2^ to 5% (File S2 Figure S6A) and 49% among studies that had a sample size of less than 100 (File S2, Figure S6C). Moreover, studies that did not report the class or form of ART used seem to have contributed minimally to heterogeneity (File S2, Figure S6E). Likewise, for ALP in ART-exposed individuals, Asia and case control studies and studies that did not specify the form or class of ART were the contributors to the observed heterogeneity, as shown in File S2, Figure S7A,B,E.

## 4. Discussion

This systematic review and meta-analysis examined data from 26 clinical studies investigating the effects of HIV infection and ART on liver function among PLWH. Our results revealed a significant elevation in AST and ALT among PLWH who were ART-naïve compared with HIV-negative individuals, indicating that HIV infection itself may play a role in hepatic impairment [32,47,49,50,51,52,54,55,60]. However, we noted no significant differences in ALP in ART-naïve individuals when compared with HIV-negative individuals [32,34,35,49,60].

Additionally, we noted a significant increase in all liver enzymes, including AST, ALT, and ALP, in PLWH undergoing ART when compared with HIV-negative individuals. The findings demonstrate that ART, although crucial for HIV infection management, is associated with hepatotoxicity [35,36,37,46,47,49,51,54,56,59,61,62]. The notable increase in AST and ALT levels among ART–exposed individuals is supported by previous studies [32,66,67]. More recently, Gbolahan and colleagues observed substantial elevations in AST and ALT levels in PLWH undergoing HAART and those who were pre-HAART compared with HIV-negative controls [61]. The same trend was previously reported by another study, which found that individuals using ART had significantly increased AST and ALT and ALP levels [36,62]. Altogether, these results support the correlation between ART exposure and hepatic dysfunction. These suggest that ART may exacerbate liver dysfunction among PLWH.

Other researchers have shown increased ALT in PLWH compared with HIV-negative individuals, and this was reportedly observed during ART exposure [64]. This is supported by recent evidence, which showed that AST and ALT were increased as the duration of ART exposure increased [37]. Others suggest that an increase in the level of the liver enzymes depends on the baseline CD4 count when ART is first initiated. This is supported by Deshmukh et al., 2024 [63], who reported a significant yet negative correlation between CD4 count and elevated liver enzymes, suggesting that the lower the CD4 count, the higher the AST and ALT in PLWH exposed to ART. Previous evidence has also shown an association between liver dysfunction, especially in ART-exposed PLWH, and age, suggesting that as PLWH age, the risk of liver dysfunction also increases [68].

Different findings were reported in another study, showing no significant change in AST, ALT, or ALP levels between ART and ART-naïve individuals, suggesting that specific regimens may have specific effects on liver function, with some promoting safer liver function while others having null effects [69]. This is partly supported by our subgroup analysis, which showed that the class of ART has no effect on the ALP in ART-naïve. Although our findings showed an association between ART exposure and liver dysfunction, this might be exacerbated by HIV infection. The observed elevation in liver enzymes among PLWH who were ART-naïve when compared with HIV-negative individuals further supports the notion that HIV infection itself may induce liver dysfunction. Other studies consistently support this. For instance, Anyanwu et al. (2021) reported a significant increase in AST levels among PLWH not receiving ART [70]. However, the magnitude of elevation in the ART-naïve group was lower than that in the ART-exposed group, highlighting the potential effect of ART on liver health deterioration. Although it is difficult to distinguish the hepatic dysfunction arising from HIV infection or ART exposure, it is crucial in clinical settings to understand this complex interaction to make a concrete distinction. A recent study in liver cancer therapeutics has shown that targeting metabolic pathways and key regulators, such as STAM binding protein-like 1 (STAMBPL1), may offer novel strategies to alleviate the risk of hepatocellular carcinoma among those with chronic liver injury, including those affected by HIV and long-term HIV exposure [71]. In a preclinical study, a 12-month exposure to NRTIs led to liver oxidative damage concomitant with mitochondrial DNA loss [23]. This results in mutation, which impairs the function of the mitochondrial electron transport chain, thus reducing ATP synthesis. This lack of energy impairs hepatocyte function [72,73].

**Figure 6:**
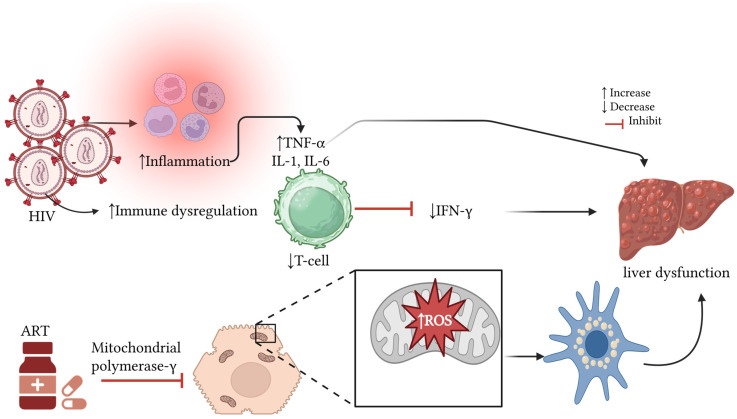
Pathways by which HIV infection and ART contribute to liver dysfunction [81]. Created using Biorender. INF-γ: interferon-gamma; TNF-α: tumor necrosis factor alpha; IL-6: interleukin-6; ROS: reactive oxygen species; ART: antiretroviral therapy; HIV: human immunodeficiency virus.

Based on the observed findings, the evidence suggests that the elevations in these liver enzymes can be attributed to HIV infection and the effects of ART exposure. Hepatocyte oxidative stress is reported to be an inducer of hepatic dysfunction in PLWH [19]. Some of the mechanisms by which ART increases these liver enzymes seem to be associated with immune reconstitution inflammatory syndrome (IRIS) and directly through drug-induced liver damage [74,75]. Chronic HIV infection induces liver dysfunction, and this is mediated through persistent inflammation and a reduction in hepatoprotective factors like interferon gamma (IFN-γ), which results in liver fibrosis, as shown in Figure 6 [64,76]. As PLWH have reduced CD4+ T-cells and dysregulated immune responses, this can suppress IFN-γ, thus resulting in fibrosis [76]. HIV infects the gut lymphoid tissue, damaging the intestinal epithelium and increasing permeability. This allows the translocation of bacterial lipopolysaccharides (LPS) to the hepatocytes, thus activating Kupffer cells [76]. It can also infect Kupffer cells and hepatocytes indirectly through inflammatory mediators, leading to elevated liver enzymes [77,78]. The HIV glycoprotein (gp120) binds to chemokine receptors on hepatic stellate cells, activating metabolic pathways that promote the generation of reactive oxygen species (ROS) and collagen production; altogether, these promote liver fibrosis [64,76].

On the other hand, the ART-associated liver enzyme elevation mechanisms are complex; these encompass mitochondrial toxicity, direct hepatotoxicity from ART, and alterations in bile acid metabolism [79]. NRTI regimens are known to induce mitochondrial dysfunction, resulting in oxidative stress and hepatocellular damage [23]. Similarly, NNRTIs, such as nevirapine, ritonavir, and lopinavir, impair liver enzyme metabolism, resulting in elevated AST, ALT, and ALP levels. Likewise, efavirenz contributes to hepatic steatosis, resulting in liver enzyme elevation [80]. More recently, our team found that ART exposure in pregnant women with pre-eclampsia promotes immune activation and inflammation, resulting in elevated liver enzymes and hepatotoxicity [81].

Our findings imply that there is a need for regular monitoring of liver function among PLWH undergoing ART. Recent evidence suggests that combining tenofovir, lamivudine, and dolutegravir may reduce liver function relative to traditional regimens [64]. A routine evaluation of AST, ALT, and ALP levels should be incorporated into primary and secondary healthcare clinical practice to identify ART-induced hepatic impairment early and develop strategies to curb these dysfunctions. This could assist in providing effective treatment for HIV with fewer hepatic side effects.

### Strengths and Limitations

Several strengths and limitations should be acknowledged. First, this study adhered to the PRISMA guideline of reporting and is registered with the Open Science Framework registry to ensure transparency. Of the included studies, 15 (58%) were regarded as high quality and low risk of bias, while 11 (42%) were classified as moderate quality and risk of bias. These suggest that the overall quality of the included studies was good. It is worth noting that these studies exhibited significant heterogeneity, which seems to have influenced the overall effect estimates in AST, especially in ART-naïve patients when compared with HIV-negative patients. However, a detailed subgroup analysis was conducted to find the potential sources, showing that larger samples yielded more consistent outcomes. While the form or class of ART used was not reported in all studies, we were able to conduct a subgroup analysis to find the sources of the observed heterogeneity. Many studies were cross-sectional and case studies, with a lack of longitudinal studies to assess the long-term effect of ART on these liver function tests. Although the overall sample size was sufficient, it is important to acknowledge that individual studies’ sample sizes ranged from 20 to 400 participants. While HIV is more prevalent in South Africa, among the analyzed studies, none were conducted in this country. Additionally, only 4 studies were conducted on female participants.

## 5. Conclusions

This study demonstrates that both HIV infection and ART exposure are associated with elevated liver enzymes (AST, ALT, and ALP). ART-exposed patients showed an elevation in AST, ALT, and ALP enzymes. In ART-naïve individuals, there was a pronounced increase in AST and ALT without an effect on ALP compared with HIV-negative individuals.

### Recommendation and Future Perspectives

Based on the findings from this study, we recommend that future clinical studies focus on newer ART regimens, increase follow-up, and report the exact form of ART regimens used to ascertain their contribution to liver dysfunction. We also recommend that, in clinical settings, the liver function should be taken into consideration when ART is first initiated and should be regularly monitored in those exposed to ART so that any dysfunction can be identified early and further controlled or prevented. Regular monitoring of liver enzymes through tests alongside CD4 counts and viral load is recommended, especially for those exposed to HAART. This initiation will help in deciding on the discontinuation of ART regimens if it is associated with increased toxicity. Our findings warrant individualized ART selection, considering pre-existing hepatic dysfunctions and drug–drug interactions. Additionally, conducting longitudinal studies to evaluate liver function in ART-naïve and ART-exposed patients may be necessary in HIV-prevalent communities. Therefore, we recommend that future studies focus more on female participants, especially in HIV-prevalent countries like South Africa, to better inform the health care system in improving the health of these populations, as the findings obtained in this study might not be translated to such prevalent populations. Additionally, patient education on liver health and potential ART-related side effects may be important to reduce hepatic complications associated with ART exposure. These measures can improve liver health management, enhance patient outcomes, and improve the quality of life of PLWH.

## Data Availability

All secondary data analyzed in this study can be found in the Supplementary Files.

## Supplementary Materials

The following supporting information can be downloaded at https://www.mdpi.com/article/10.3390/ph18070955/s1, Supplementary File S1: PRISMA checklist. Supplementary File S2: Figure S1: Result of the sensitivity analysis based on the trim and fill test. A: AST in ART-naïve compared with HIV negative. B: ALT in ART-naïve compared with HIV negative. C: AST in ART compared with HIV negative. D: ALT in ART compared with HIV negative; Figure S2: Subgroup analysis on AST in ART-naïve compared to HIV-negative individuals. A: The level of AST in PLWH who were ART-naïve versus HIV negative, based on study design. B: ART-naïve versus HIV negative on AST based on continent. C: AST levels in PLWH who are ART-naïve versus HIV negative, based on sample size. D: AST levels in ART-naïve versus HIV negative, based on gender distribution. E: AST levels in ART-naïve versus HIV negative, based on class of ART; Figure S3: Subgroup analysis on AST in PLWH exposed to ART compared with HIV-negative individuals. A: AST in PLWH exposed to ART compared with HIV-negative individuals based on study design. B: AST in PLWH exposed to ART compared with HIV-negative individuals based on the continent of publication. C: AST in PLWH exposed to ART compared with HIV-negative individuals based on sample size. D: AST in PLWH exposed to ART compared with HIV-negative individuals based on gender distribution. E: AST in PLWH exposed to ART compared with HIV-negative individuals based on the class of ART; Figure S4: Subgroup analysis on ALT in ART-naïve compared with HIV-negative individuals. A: ALT level in PLWH on ART-naïve group compared with HIV negative, based on study design. B: Levels of ALT in ART-naïve PLWH compared with HIV negative, based on the continent of publication. C: ALT level in PLWH on ART-naïve compared with HIV negative, based on sample size. D: ALT levels in PLWH on ART-naïve compared with HIV negative, based on gender distribution. E: ALT levels in PLWH on ART-naïve group compared with HIV negative based on class of ART regimens; Figure S5: Subgroup analysis on ALT among PLWH on ART compared with HIV-negative individuals. A: ALT among PLWH on ART compared with HIV-negative individuals based on study design. B: ALT among PLWH on ART compared with HIV negative individuals based on continent. C: ALT among PLWH on ART compared with HIV-negative individuals based on sample size. D: ALT among PLWH on ART compared with HIV-negative individuals based on gender distribution. E: ALT among PLWH on ART compared with HIV-negative individuals based on the class of ART regimens; Figure S6: Subgroup analysis showing the effect of different factors on ALP in PLWH who are ART-naïve compared with HIV negative. A: ALP levels in PLWH who are ART-naïve compared with HIV-negative individuals, based on study design. B: ALP level in PLWH who are ART-naïve vs. HIV negative, based on the continent of publication. C: ALP levels in PLWH who are ART-naïve compared with HIV negative, based on sample size. D: ALP levels in PLWH who are ART-naïve compared with HIV negative, based on gender. E: ALP levels in PLWH who are ART-naïve compared with HIV negative, based on the class of ART regimens; Figure S7: Subgroup analysis showing the effect of different factors on ALP in PLWH on ART compared with HIV negative. A: ALP levels in PLWH on ART compared with HIV-negative individuals, based on study design. B: ALP levels in PLWH on ART compared with HIV-negative individuals in terms of the continent of publication. C: ALP levels in PLWH on ART compared with HIV-negative individuals based on gender. D: ALP levels in PLWH on ART compared with HIV-negative individuals based on sample size. E: ALP levels in PLWH on ART compared with HIV-negative individuals based on class of ART regimens; Table S1: General overview of characteristics of included studies; Table S2: Quality assessment of cohort studies; Table S3: Quality assessment of cross-sectional studies; Table S4: Quality assessment of case control studies; Table S5: Sensitivity analysis using one study exclusion at a time on AST in PLWH compared to HIV negative; Table S6: Sensitivity analysis using one study exclusion at a time on ALT in PLWH compared to HIV negative.

## Abbreviations

AIDS	acquired immunodeficiency virus
ALP	alkaline phosphatase
ALT	alanine aminotransferase
ART	antiretroviral therapy
ARV	antiretroviral
AST	aspartate aminotransferase
CD4	cluster of differentiation 4
CI	confidence interval
DTG	dolutegravir
HAART	highly active antiretroviral therapy
HBV	hepatitis B Virus
HCV	hepatitis C Virus
HIV	human immunodeficiency virus
MeSH	Medical Subject Headings
NNRTI	non-nucleoside reverse transcriptase inhibitors
NOS	Newcastle–Ottawa Scale
NRTI	nucleoside reverse transcriptase inhibitor
PICO	population, intervention, comparator, outcome
PLWH	people living with HIV
PRISMA	Preferred Reporting Items for Systematic Reviews and Meta-Analyses
SD	standard deviation
SEM	standard error of mean
SMD	standardized mean difference
