# Supplementary material for: HIV Infection and Antiretroviral Therapy Impair Liver Function in People Living with HIV: Systematic Review and Meta-Analysis
Source: Pharmaceuticals (Basel). 2025 Jun 25;18(7):955. doi: 10.3390/ph18070955 (PMC12300453; doi:10.3390/ph18070955)
Supplement: Supplementary file 1 [file pharmaceuticals-18-00955-s001.zip › File S2_Supplementary figures and tables.pdf]

Supplementary File S2

HIV Infection and Antiretroviral Therapy Impair Liver Function in People Living with HIV: Systematic Review and Meta-Analysis

Kay-Lee E Strauss [1], Wendy N Phoswa [1], Sidney Hanser [2] and Kabelo Mokgalaboni [1,*]

[1]Department of Life and Consumer Sciences, College of Agriculture and Environmental Sciences, University of South Africa, Florida Campus, 1710, 68188242@mylife.unisa.ac.za; phoswwn@unisa.ac.za; mokgak@unisa.ac.za

[2]Department of Physiology and Environmental Health, University of Limpopo, Sovenga 0727, South Africa; sidney.hanser@ul.ac.za

*Correspondence: mokgak@unisa.ac.za

Supplementary File S2

Table S1. General overview of characteristics of included studies

Author, year and reference	Country	Study design	Population Size	Age	Treatments and duration	Baseline CD4 of the ART/ART-naïve group Mean ± SD	Male In the ART group n (%)	Effect on liver enzymes
**Abdulmumin et al., 2024** [1]	Nigeria	Cross-sectional	100 people living with HIV (PLWH) on HAART<br>100 HIV-negative individuals	18–50	Tenofovir, Lamivudine, and Efavirenz; (TDF+3TC+EFV) (70%) and 30% were on Zidovudine, Lamivudine, and Efavirenz for 4–12 years	NR	25 (25)	HAART significantly increased ALT and AST levels compared to HIV-negative individuals, with no effect on ALP.
**Abriba et al., 2024** [2]	Nigeria	Case-control	17 PLWH on ART<br>15 PLWH not on ART<br>12 HIV-negative	18–65	NR	NR	23 (45)	Significant increase in ALT, ALP, and AST in ART compared to HIV negative. However, no changes in ALP despite an increased AST and ALT in ART-naïve compared to HIV-negative.
**Deshmukh et al., 2024** [3]	India	Case-control	150 PLWH on ART<br>50 HIV-negative	16-50	NR	≤ 200<br>20–500<br>≥ 500 cells/µl	NR	Significant increase in AST and ALT in PLWH on ART compared to the HIV-negative.
**Odegbemi et al., 2024** [4]	Nigeria	Cross-sectional	120 PLWH on ART<br>50 HIV-negative	18–65	Tenofovir/Lamivudine, Dolutegravir (TLD)	NR	52 (43.3)	Significant decrease in AST without change in ALT in ART compared to HIV-negative.
**Tamuno–Boma et al., 2023** [5]	Nigeria	Cross-sectional	83 pregnant women living with HIV on ART<br>82 non-pregnant women living with HIV on ART<br>84 pregnant HIV-negative<br>81 non-pregnant HIV negative	15–60	HAART for over 6 months	NR	0 (0)	Significant increase in ALP, ALT, and AST levels in both groups compared to the control group.

Supplementary File S2

Author, year and reference	Country	Study design	Population Size	Age	Treatments and duration	Baseline CD4 of the ART/ART-naïve group Mean ± SD	Male In the ART group n (%)	Effect on liver enzymes
**Gbolohan et al., 2023** [6]	Nigeria	Cross-sectional	59 PLWH on HAART<br>34 PLWH who are pre-HAART.<br>50 HIV-negative	NR	HAART	NR	41 (44)	Compared to healthy individuals, there was a significant increase in AST and ALT levels among PLWH on HAART and pre-HAART.
**Gospel et al., 2023** [7]	Nigeria	Cross-sectional Comparative	100 PLWH on ART<br>100 HIV without ART<br>100 HIV-negative	20 –70	HAART for 7 months	NR	NR	Significant increases in ALT and ALP levels and decreased AST in ART and ART-naïve compared to HIV-negative individuals.
**Mutuma et al., 2023** [8]	Kenya	Cross-sectional	47 PLWH who are HAART adherent<br>23 HAART-naïve PLWH<br>51 HIV-negative	18–60	HAART (Tenofovir Disoproxil Fumarate, Lamivudine, and Efavirenz) for more than 6 months	≥ 500 CD4+ cells/ µl<br>350 – 499 cells/ µl 200 – 349 cells/ µl<br>< 200 cells/ µl	32 (68)	Significant increase in AST and ALP in ART and ART naïve compared to HIV negative. ALT increased in ART without a change in ATR-naïve.
**Younis et al., 2022** [9]	Lybia	Case-control	101 PLWH on ART<br>21 ART-naïve<br>70 HIV-negative	20–45	ARV triple combination (FTC + TDF + EFV; FTC+ TDF + LPV/r, FTC+ TAF + EVG/c or RAL) more than 24 weeks	648.3 ± 400.5<br>884.5 ± 369.8	NR	Significant increase in ALT and AST in ART and ART-naïve compared to HIV -negative.
**Ezeugwunne et al., 2021** [10]	Nigeria	Case-control	45 PLWH on ART<br>26 PLWH not on ART<br>26 HIV-negative	18–60	Lamivudine, Stavudine, and Nevirapine	616.16±359.22<br>390.27±117.15	31 (68.9)	Significant increase in ALT without change in AST and ALP when ART and ART naïve are compared to HIV-negative.
**Ambad et al., 2021** [11]	India	Cross-sectional comparative	20 PLWH-naïve who further receive ART after 6 months.<br>20 HIV-negative	15–60	On HAART for 6 months	NR	0 (0)	Significant increase in ALP, AST, and ALT levels after 6 months of ART treatment in PLWH compared to HIV-negative individuals.
**Ikekpeazu et al., 2019** [12]	Nigeria	Comparative cross-sectional	30 pregnant women living with HIV on HAART	20–40	Nevirapine-based HAART	NR	0 (0)	Significant increase in ALP and ALT activities in PLWH compared

Supplementary File S2

Author, year and reference	Country	Study design	Population Size	Age	Treatments and duration	Baseline CD4 of the ART/ART-naïve group Mean ± SD	Male In the ART group n (%)	Effect on liver enzymes
			30 pregnant women living with HIV not on HAART<br>30 HIV-negative					to HIV-negative individuals in all trimesters.
**Quaye et al., 2019** [13]	Ghana	Observational cross-sectional	105 PLWH on ART<br>77 ART-naïve PLHIV<br>60 HIV-negative	36.05–48.86	Lamivudine (3TC), Zidovudine (AZT), Efavirenz (EFV), Nevirapine (NVP), Atazanavir (ATV), and Lopinavir (LPV for 3.7 years	< 200<br>200–350<br>> 500	45 (43)	PLWH showed significantly elevated ALP and AST without changes in ALT compared to HIV–negative.
**Emokpae et al., 2018** [14]	Nigeria	Comparative cross-sectional	50 PLWH on HAART<br>50 PLWH no ART<br>50 HIV-negative	33.53–40.60	HAART for at least 4 months	537.64±49.38<br>352.10±42.86<br>939.20±23.67	6 (16)	Significant increase in AST, ALT, and ALP in ART, ART-naïve compared to HIV-negative
**Olisekodiaka et al., 2018** [15]	Nigeria	Comparative cross-sectional	40 PLWH on ART<br>40 ART-naive<br>40 HIV-negative	18–55	HAART for at least 6 months	467.3±19.4<br>188.5 ± 21.7<br>886.6± 12.6	20 (50)	Significant increase in AST and ALT in ART and ART-naïve compared to HIV-negative
**Ashakarin et al., 2018** [16]	India	Cross-sectional case-control	90 PLWH<br>90 HIV-negative	20–60	NR	NR	37 (41)	Significant increase in AST, ALT, and ALP levels in PLWH on ART compared to HIV-negative individuals.
**Agbecha & Ikyernum 2018** [17]	Nigeria	Case-control	20 PLWH on ART<br>20 ART-naïve PLWH<br>20 HIV-negative	18–60	NR	451.20±229.18	NR	Significant increase in ALT without changes in AST and ALP in ART compared to HIV–negative. Significant increase in AST, ALT, and ALP in ART-naïve compared to HIV-negative.
**Aniagolu et al., 2017** [18]	Nigeria	Cohort	43 PLWH who are HAART naïve (given ART after 8 months)<br>20 HIV-negative	NR	Combivir N used as HAART	247± 71<br>319±139	NR	Significant increase in ALT and AST levels following 8 months of treatment.

Supplementary File S2

Author, year and reference	Country	Study design	Population Size	Age	Treatments and duration	Baseline CD4 of the ART/ART-naïve group Mean ± SD	Male In the ART group n (%)	Effect on liver enzymes
					(Zidovudine, Lamivudine and Nevirapine) for 4 and then 8 months			
**Ebot et al., 2015** [19]	Cameroon	Cross-sectional	100 PLWH on ART<br>100 ART-naïve individuals<br>100 HIV-negative	25.86–49.94	On the first line ART regimen for six months	435.5 ± 45.2<br>352.1 ± 28.7	96 (32)	Significant increase in AST and ALT without change in ALP activities compared to ART and ART-naïve, are compared to HIV-negative.
**Prathinia et al., 2015** [20]	India	Comparative cross-sectional	200 PLWH no ART<br>200 HIV-negative	19.01–61.30	None	NR	118 (59)	Significant increase in serum AST and ALT in PLHIV compared to HIV-negative
**Nwosu et al., 2015** [21]	Nigeria	Comparative cross-sectional	20 PLWH on ART<br>20 HIV–negative	53	ART for 1–10 months	NR	9 (45)	Significant decrease in AST and ALT and increased ALP in PLWH on ART compared to HIV-negative individuals.
**Ayelagbe et al., 2014** [22]	Nigeria	Comparative cross-sectional	45 PLWH on HAART<br>40 HAART-naïve<br>40 HIV-negative.	18.27–55.65	HAART<br>Didanosine + Emtricitabine or Lamivudine + Nevirapine or Efavirenz<br>Zidovudine + Lamivudine + Nevirapine or Efavirenz<br>Stavudine + Lamivudine + Nevirapine or Efavirenz for 6 months.	NR	NR	Significant increase in AST, ALT, and ALP in HAART compared to HIV-negative. Significant increase in AST and ALT without changes in ALP in HAART-naïve compared to HIV-negative.
**Abubakar et al., 2014** [23]	Nigeria	Cross-sectional comparative	25 PLWH on ART<br>25 PLWH without ART<br>25 HIV-negative individuals	20–39	2 NRTIs + NNRTIs, 2 NRTIs + A boosted protease inhibitor (Indinavir or Ritonavir) for 1 year or less	516.28±37.51	16 (64)	ART significantly reduced ALP compared to the non-ART group. No differences in AST and ALT in ART and no-–ART groups.

Supplementary File S2

Author, year and reference	Country	Study design	Population Size	Age	Treatments and duration	Baseline CD4 of the ART/ART-naïve group Mean ± SD	Male In the ART group n (%)	Effect on liver enzymes
**Ibeh et al., 2013** [24]	Nigeria	Prospective Cohort	50 PLWH on winniecure ART<br>100 HIV-negative	17 and above	Winniecure ART for 12 weeks	50–200<br>200–350 cells/μL	24 (48)	A significant increase in ALP, ALT, and AST levels following the administration of ART was observed when compared to HIV-negative individuals.
**Analike et al., 2008** [25]	Nigeria	Cross-sectional	100 PLWH on ART<br>50 PWLH not on ART<br>100 HIV-negative	18-55	Combination of Zidovudine, Lamivudine, and Nevirapine for six months	NR	NR	A significant increase in AST, ALT, and ALP in ART compared to HIV-negative patients.
**Betuine et al., 2007** [26]	Brazil	Prospective cohort	45 pregnant women living with HIV on ART<br>12 HIV-negative females	16–43	Zidovudine, and Triple T (ZDV + 3TC + NFV)	NR	0 (0)	No significant difference in AST and ALT levels in pregnant women living with HIV on ART compared to HIV-negative women.

PLWH: Pepple living with HIV, TDF: Tenofovir Disoproxil Fumarate, EFV: Efavirenz, NR: Not Reported, HAART: Highly Active Antiretroviral Therapy, ALT: Alanine Aminotransferase, AST: Aspartate Transaminase, ALP: Alkaline Phosphatase, HIV: Humam Immunodeficiency Virus, ART: Antiretroviral Therapy, TLD: Tenofovir/Lamivudine, Dolutegravir, ARV: Antiretroviral, NRTI: Nucleoside reverse transcriptase inhibitor, NNRTI: Non-nucleoside reverse transcriptase inhibitors.

Supplementary File S2

Quality Assessment of included by Newcastle-Ottawa Scale (NOS)

Table S2: Quality assessment of cohort studies

	**Selection**				**Comparability**	**Outcome**			**Score**	**Quality**	**ROB**
	Representativeness of exposed cohort	Selection of non-exposed cohort	Ascertainment of exposure	Demonstration that outcome of interest was not present at the start of study	Comparability of cohorts on the basis of the design or analysis controlled for confounders	Assessment of outcome	Was follow-up long enough for outcomes to occur	Adequacy of follow-up cohorts			
Angiagolu et al., 2017	★	★	★	☆	★	☆	★	★	6	Moderate	Moderate
Betuine et al., 2007	★	★	★	★	☆	☆	★	★	6	Moderate	Moderate
Ibeh et al., 2013	★	★	★	☆	★	☆	★	★	6	Moderate	Moderate

ROB: Risk of bias

Supplementary File S2

Table S3 : Quality assessment of cross-sectional studies

	Selection				Comparability	Outcome		Score	Quality	ROB
	Representativeness of the sample	Sample size	Non-included subjects	Ascertainment of exposure	Based on design and analysis	Assessment of outcome	Statistical test			
Abdulmumin et al., 2024	★	☆	★	★	★★	★	★	7	High	Low
Adubakar et al., 2014	★	☆	★	★	★★	☆	★	6	Moderate	Moderate
Ambad et al., 2021	★	☆	★	★	★	☆	★	5	Moderate	Moderate
Ashakarin et al., 2018	★	☆	★	★	★	☆	★	5	Moderate	Moderate
Ebot et al., 2025	★	☆	★	★	★	★	★	7	High	Low
Gbolohan et al., 2023	★	★	★	★	★★	★	☆	7	High	Low
Gospel et al., 2023	★	★	★	★	★★	☆	★	7	High	Low
Mutuma et al., 2023	★	☆	★	★	★★	★	★	7	High	Low
Odegbemi et al., 2025	★	★	★	★	★★	★	☆	7	High	Low
Quaye et al., 2019	★	☆	☆	★	★★	☆	★	6	Moderate	Moderate
Tamuno-Boma et al., 2023	★	★	★	★	★★	☆	★	7	High	Low
Ikekpeazu et al., 2019	★	☆	★	☆	★★	☆	★	5	Moderate	Moderate
Emokpae et al., 2018	★	☆	★	★	★★	★	★	7	High	Low
Olisekodiaka et al., 2018	★	☆	★	★	★★	★	★	7	High	Low
Prathima et al., 2015	★	☆	★	★	★	★	★	6	Moderate	Moderate
Nwosu et al., 2015	★	☆	★	★	★★	★	★	7	High	Low
Ayelagbe et al., 2014	★	☆	★	★	★★	★	☆	6	Moderate	Moderate
Analike et al., 2008	★	☆	★	★	★	★	★	6	Moderate	Moderate

ROB: Risk of bias

Supplementary File S2

Table S4: Quality assessment of case control studies

	Selection				Comparability	Exposure			Score	Quality	ROB
	**Is the case definition adequate?**	**Representativeness of the case**	**Selection of controls**	**Definition of controls**	**Comparability of cases and controls on the bases of the design or analysis**	**Ascertainment of exposure**	**Same method of ascertainment for cases and controls**	**Non-Response rate**			
Agbecha & Ikyernum 2018	★	★	★	★	★★	★	★	☆	8	High	Low
Ezeugwunne et al., 2021	★	★	★	★	★★	★	☆	★	8	High	Low
Abriba et al., 2024	★	★	★	★	★★	★	★	☆	8	High	Low
Deshmikh et al, 2024	★	★	★	★	★	★	★	☆	7	High	Low
Younis et al., 2022	★	★	★	★	★★	★	★	☆	8	High	Low

ROB: Risk of bias

Supplementary File S2

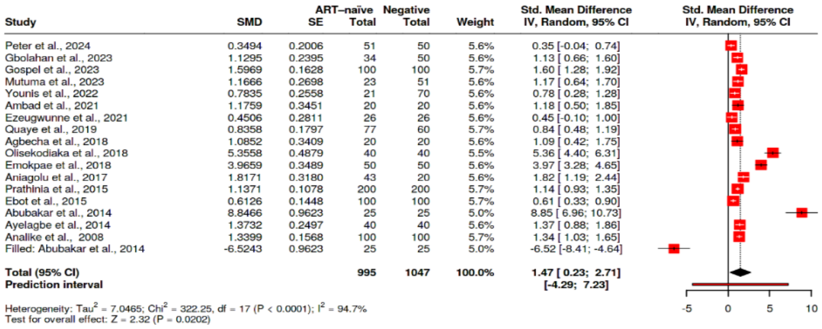

A

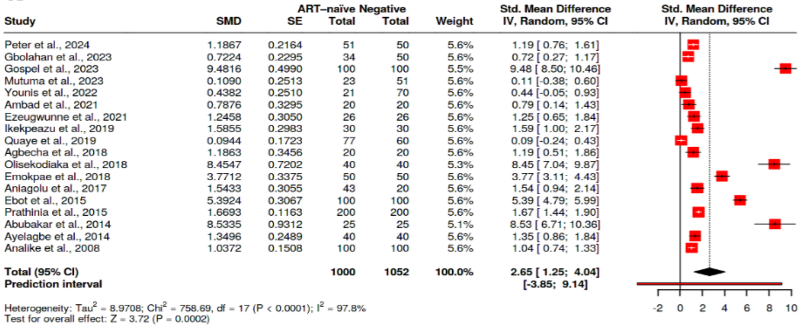

B

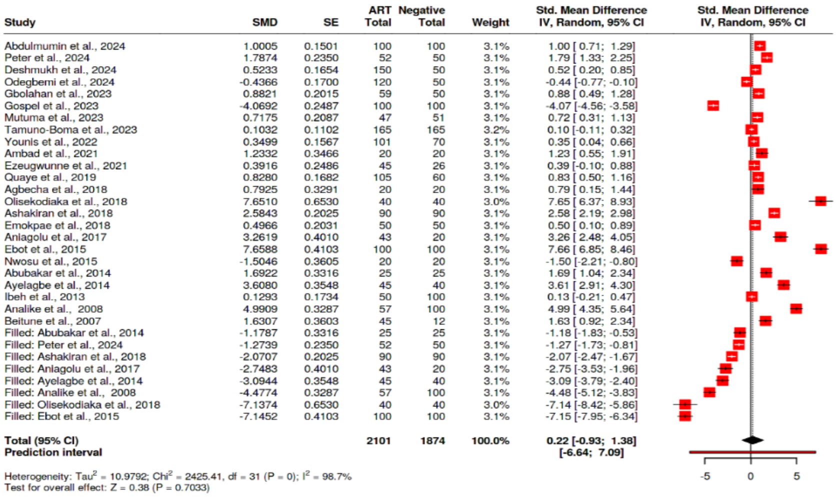

C

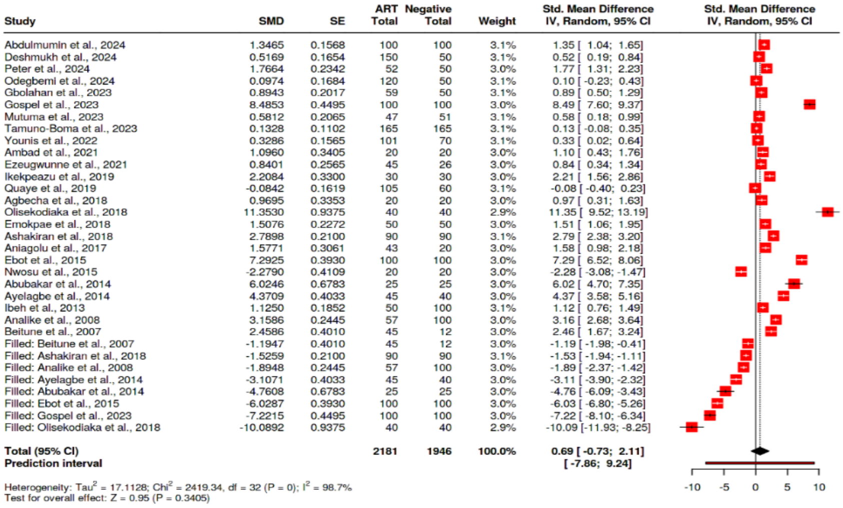

D

Figure S1: Result of the sensitivity analysis based on the trim and fill test. A: AST in ART-naïve compared with HIV negative. B: ALT in ART-naïve compared with HIV negative. C: AST in ART compared with HIV negative. D: ALT in ART compared with HIV negative.

Supplementary File S2

Table S5: Sensitivity analysis using one study exclusion at a time on AST in PLWH compared to HIV negative

**Studies**	**Effect size (SMD)**	**Lower Confidence intervals**	**Upper Confidence intervals**	**P**
Abdulmumin	1.51	0.46	2.56	<0.05
Peter	1.48	0.42	2.53	<0.05
Deshmukh	1.53	0.48	2.59	<0.05
Odegbemi	1.58	0.53	2.62	<0.05
Gbolahan	1.52	0.46	2.57	<0.05
Gospel	1.73	0.80	2.66	<0.05
Mutuma	1.53	0.47	2.58	<0.05
Tamuno-Boma	1.55	0.50	2.60	<0.05
Younis	1.54	0.49	2.59	<0.05
Ambad	1.50	0.45	2.56	<0.05
Ezeugwunne	1.54	0.49	2.59	<0.05
Quaye	1.52	0.47	2.58	<0.05
Agbecha	1.52	0.47	2.58	<0.05
Olisekodiaka	1.23	0.32	2.15	<0.05
Ashakiran	1.44	0.39	2.50	<0.05
Emokpae	1.54	0.48	2.59	<0.05
Aniagolu	1.42	0.37	2.46	<0.05
Ebot	1.22	0.32	2.12	<0.05
Nwosu	1.62	0.60	2.64	<0.05
Abubakar	1.48	0.43	2.54	<0.05
Ayelagbe	1.40	0.36	2.44	<0.05
Ibeh	1.55	0.50	2.50	<0.05
Analike	1.34	0.33	2.35	<0.05
Beitune	1.49	0.43	2.54	<0.05

Supplementary File S2

Table S6. Sensitivity analysis using one study exclusion at a time on ALT in PLWH compared to HIV negative

Studies	Effect size (SMD)	Lower CI	Upper CI	P
Abdulmumin	2.34	1.14	3.54	<0.05
Peter	2.37	1.18	3.57	<0.05
Deshmukh	2.32	1.12	3.52	<0.05
Odegbemi	2.39	1.20	3.58	<0.05
Gbolahan	2.36	1.16	3.56	<0.05
Gospel	2.03	0.96	3.11	<0.05
Mutuma	2.37	1.18	3.57	<0.05
Tamuno-Boma	2.39	1.20	3.58	<0.05
Younis	2.38	1.19	3.57	<0.05
Ambad	2.35	1.15	3.55	<0.05
Ezeugwunne	2.36	1.16	3.56	<0.05
Ikekpeazu	2.30	1.10	3.51	<0.05
Quaye	2.40	1.21	3.58	<0.05
Agbecha	2.35	1.16	3.55	<0.05
Olisekodiaka	1.94	0.98	2.91	<0.05
Emokpae	2.33	1.13	3.53	<0.05
Ashakiran	2.28	1.08	3.48	<0.05
Aniagolu	2.33	1.13	3.53	<0.05
Ebot	2.09	0.97	3.20	<0.05
Nwosu	2.48	1.35	3.62	<0.05
Abubakar	2.15	0.99	3.31	<0.05
Ayelagbe	2.21	1.02	3.40	<0.05
Ibeh	2.35	1.15	3.55	<0.05
Analike	2.26	1.06	3.46	<0.05
Beitune	2.29	1.09	3.50	<0.05

Supplementary File S2

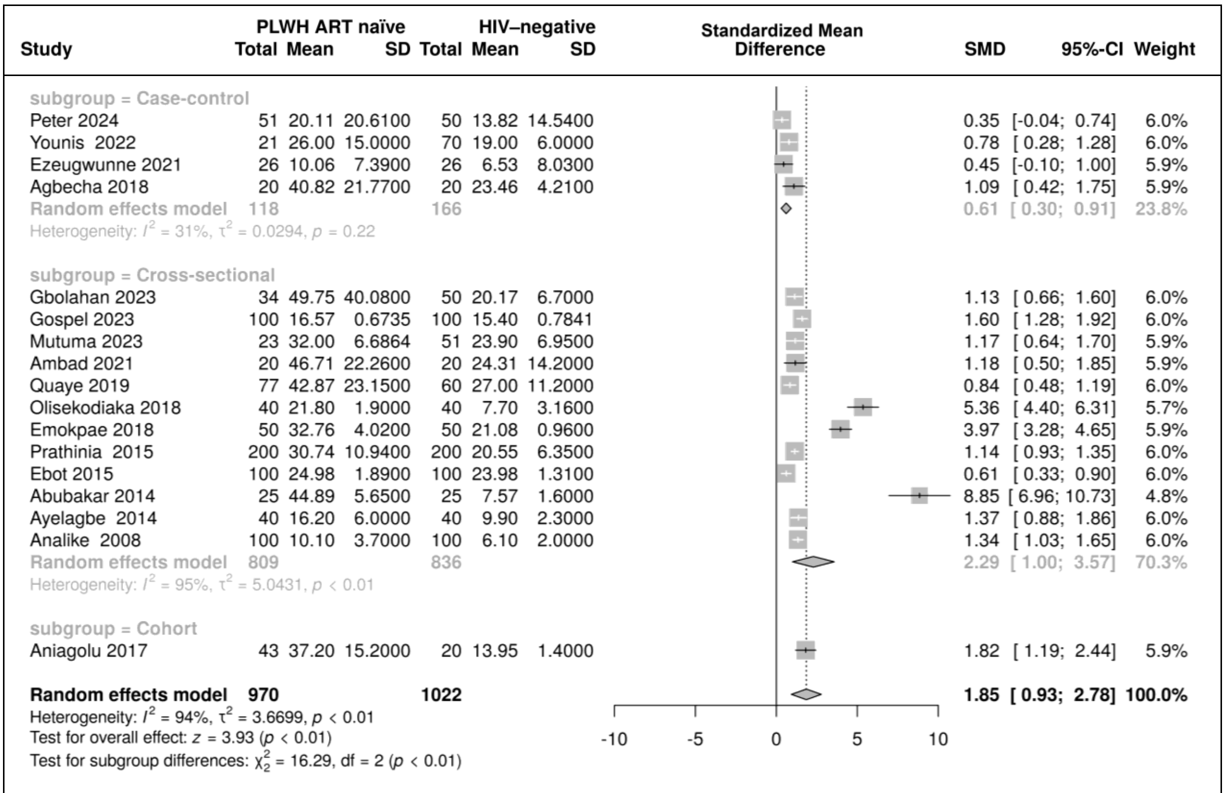

A

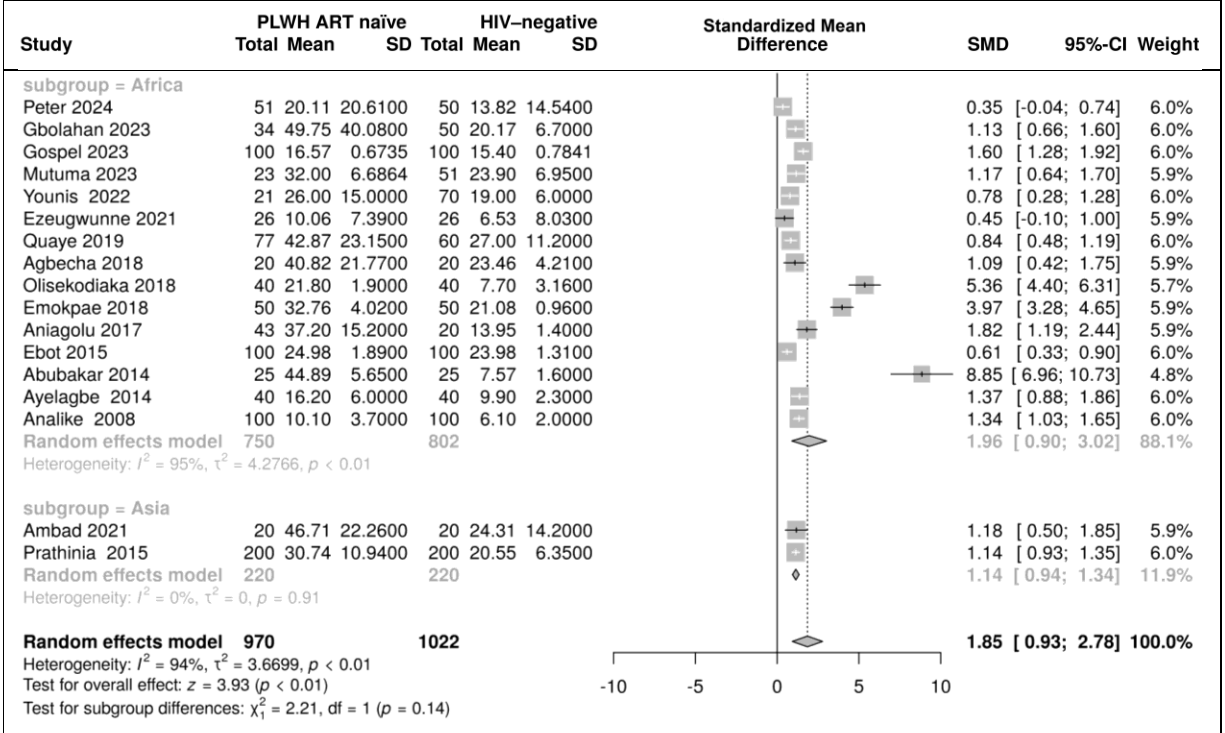

B

Supplementary File S2

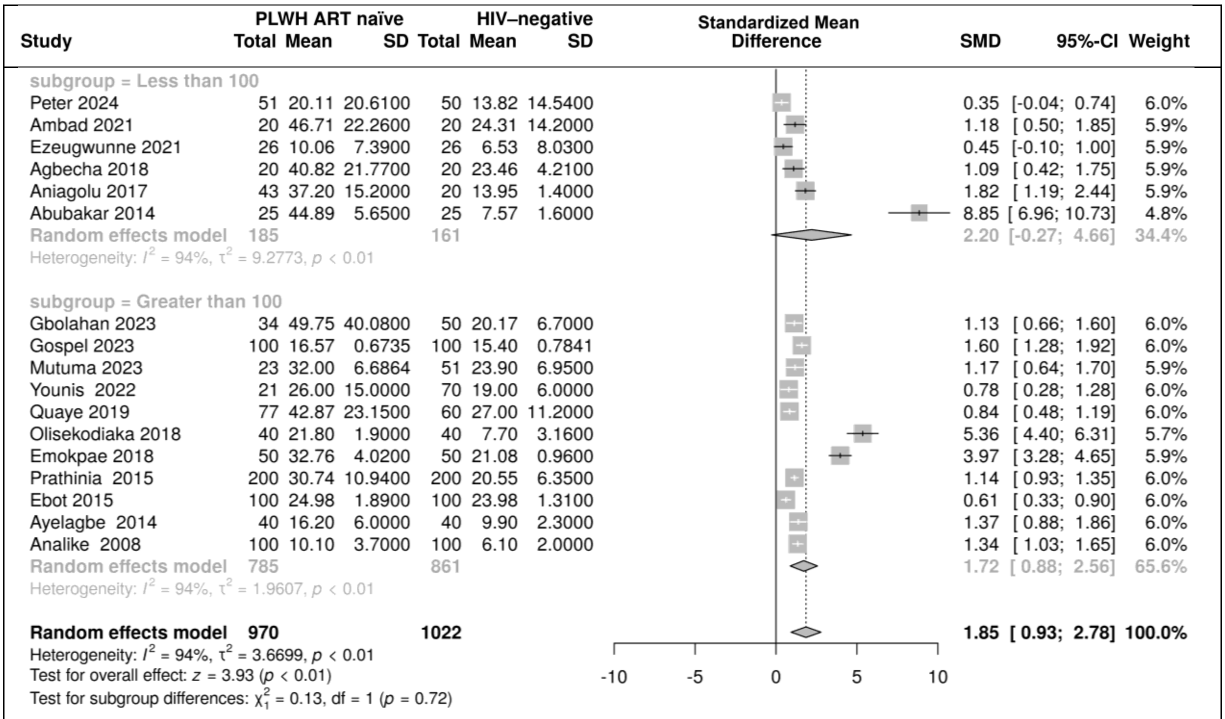

C

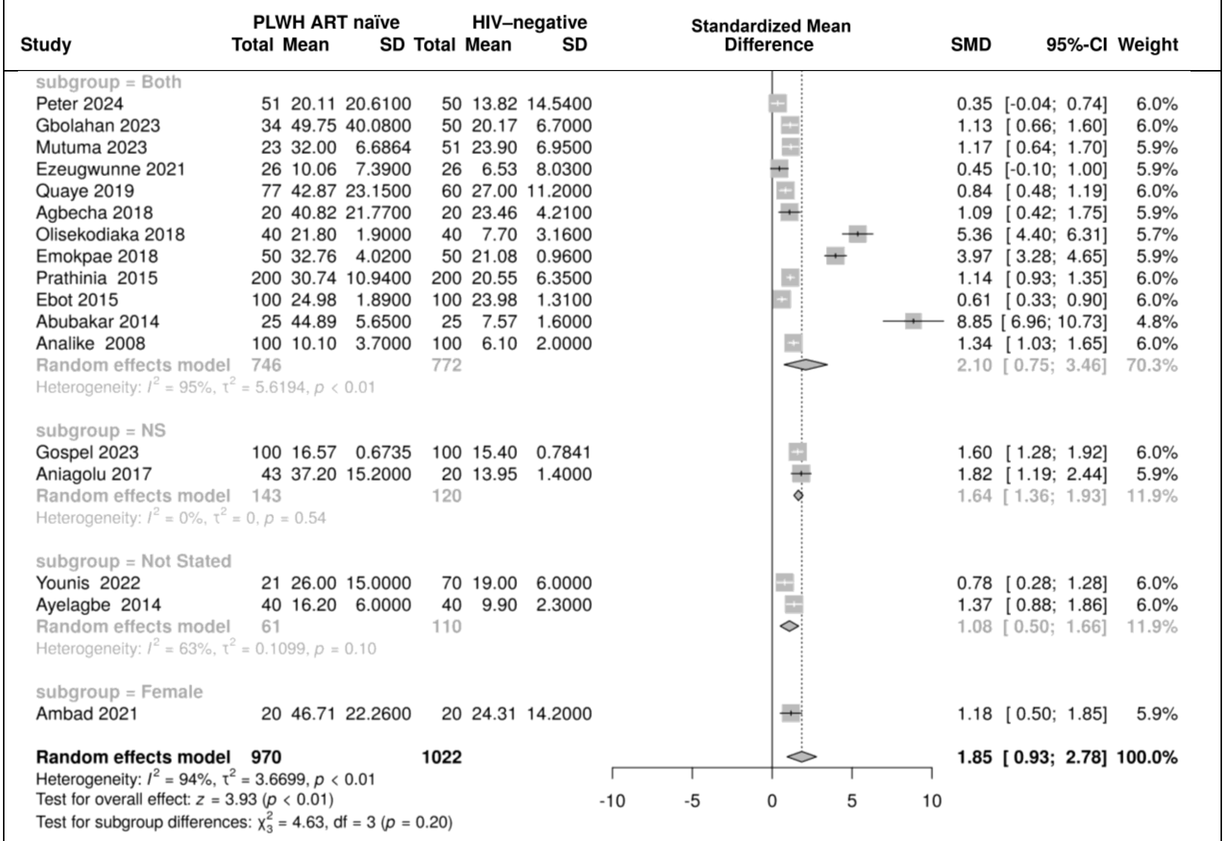

D

Supplementary File S2

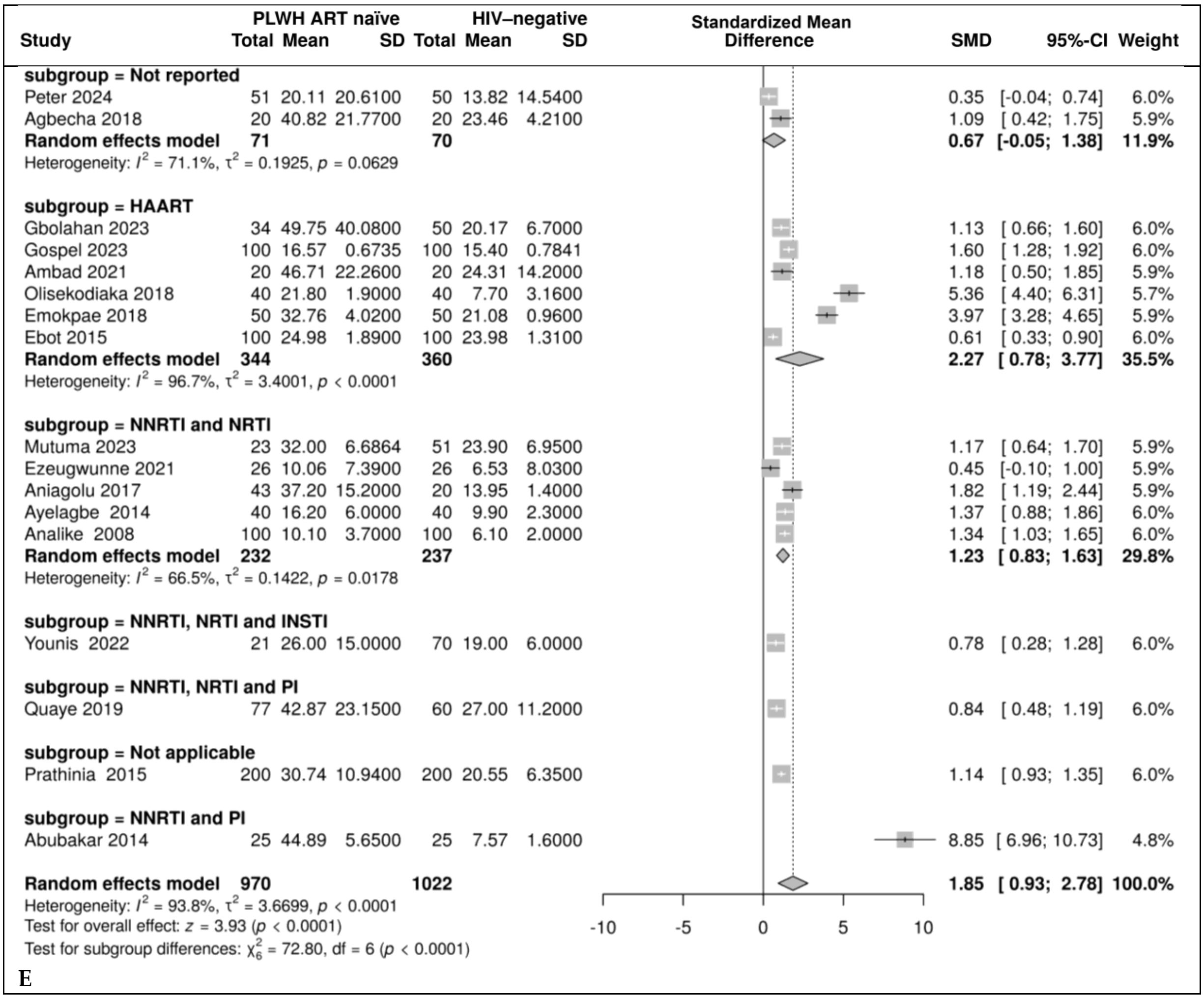

Figure S2: Subgroup analysis on AST in ART-naïve compared to HIV-negative individuals. A: The level of AST in PLWH who were ART-naïve versus HIV negative, based on study design. B: ART-naïve versus HIV negative on AST based on continent. C: AST levels in PLWH who are ART-naïve versus HIV negative, based on sample size. D: AST levels in ART-naïve versus HIV negative, based on gender distribution. E: AST levels in ART-naïve versus HIV negative, based on class of ART. The solid line shows the line of no effect, the dashed line shows the effect size, the gray block shows the weight of the study, the horizontal line crossing the gray block shows the confidence intervals, diamond plot shows the combined effect size. NRTIs: nucleoside reverse transcriptase inhibitors, NNRTIs: non-nucleoside reverse transcriptase inhibitors, NtRTI: nucleotide reverse transcriptase inhibitors, PIs: protease inhibitors, INSTIs: integrase-nucleoside strand transfer inhibitors.

Supplementary File S2

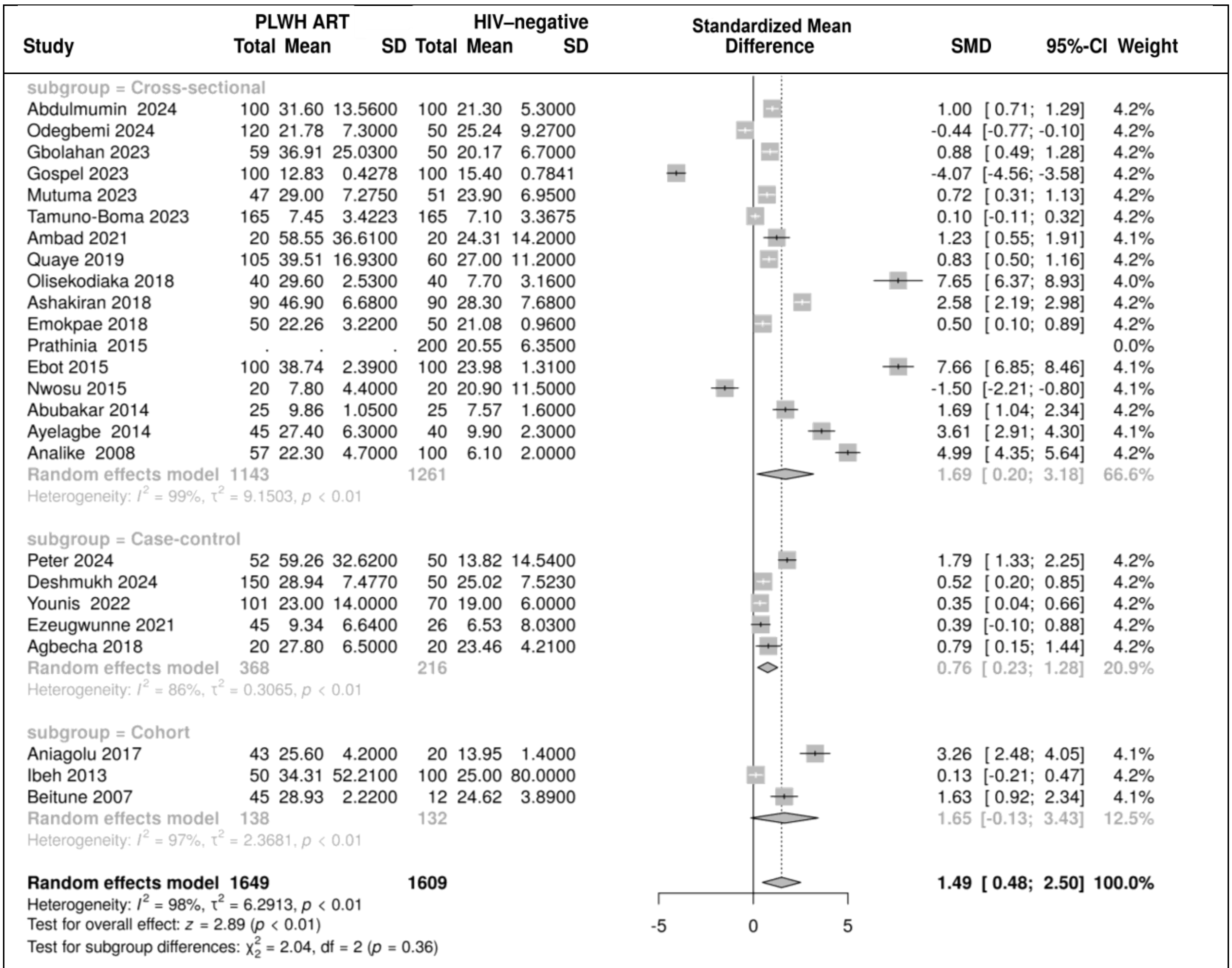

A

Supplementary File S2

Study	PLWH ART Total	Mean	SD	HIV−negative Total	Mean	SD	SMD	95%-CI	Weight
subgroup = Africa									
Abdulmumin 2024	100	31.60	13.5600	100	21.30	5.3000	1.00	[0.71; 1.29]	4.2%
Peter 2024	52	59.26	32.6200	50	13.82	14.5400	1.79	[1.33; 2.25]	4.2%
Odegbemi 2024	120	21.78	7.3000	50	25.24	9.2700	-0.44	[-0.77; -0.10]	4.2%
Gbolahan 2023	59	36.91	25.0300	50	20.17	6.7000	0.88	[0.49; 1.28]	4.2%
Gospel 2023	100	12.83	0.4278	100	15.40	0.7841	-4.07	[-4.56; -3.58]	4.2%
Mutuma 2023	47	29.00	7.2750	51	23.90	6.9500	0.72	[0.31; 1.13]	4.2%
Tamuno-Boma 2023	165	7.45	3.4223	165	7.10	3.3675	0.10	[-0.11; 0.32]	4.2%
Younis 2022	101	23.00	14.0000	70	19.00	6.0000	0.35	[0.04; 0.66]	4.2%
Ezeugwunne 2021	45	9.34	6.6400	26	6.53	8.0300	0.39	[-0.10; 0.88]	4.2%
Quaye 2019	105	39.51	16.9300	60	27.00	11.2000	0.83	[0.50; 1.16]	4.2%
Agbecha 2018	20	27.80	6.5000	20	23.46	4.2100	0.79	[0.15; 1.44]	4.2%
Olisekodiaka 2018	40	29.60	2.5300	40	7.70	3.1600	7.65	[6.37; 8.93]	4.0%
Emokpae 2018	50	22.26	3.2200	50	21.08	0.9600	0.50	[0.10; 0.89]	4.2%
Aniagolu 2017	43	25.60	4.2000	20	13.95	1.4000	3.26	[2.48; 4.05]	4.1%
Ebot 2015	100	38.74	2.3900	100	23.98	1.3100	7.66	[6.85; 8.46]	4.1%
Nwosu 2015	20	7.80	4.4000	20	20.90	11.5000	-1.50	[-2.21; -0.80]	4.1%
Abubakar 2014	25	9.86	1.0500	25	7.57	1.6000	1.69	[1.04; 2.34]	4.2%
Ayelagbe 2014	45	27.40	6.3000	40	9.90	2.3000	3.61	[2.91; 4.30]	4.1%
Ibeh 2013	50	34.31	52.2100	100	25.00	80.0000	0.13	[-0.21; 0.47]	4.2%
Analike 2008	57	22.30	4.7000	100	6.10	2.0000	4.99	[4.35; 5.64]	4.2%
Random effects model	1344			1237			1.49	[0.28; 2.71]	83.3%
Heterogeneity: $I^2 = 98\%$, $\tau^2 = 7.5548$, $p < 0.01$									
subgroup = Asia									
Deshmukh 2024	150	28.94	7.4770	50	25.02	7.5230	0.52	[0.20; 0.85]	4.2%
Ambad 2021	20	58.55	36.6100	20	24.31	14.2000	1.23	[0.55; 1.91]	4.1%
Ashakiran 2018	90	46.90	6.6800	90	28.30	7.6800	2.58	[2.19; 2.98]	4.2%
Prathinia 2015	.	.	.	200	20.55	6.3500			0.0%
Random effects model	260			360			1.45	[0.24; 2.66]	12.5%
Heterogeneity: $I^2 = 97\%$, $\tau^2 = 1.0835$, $p < 0.01$									
subgroup = America									
Beitune 2007	45	28.93	2.2200	12	24.62	3.8900	1.63	[0.92; 2.34]	4.1%
**Random effects model**	**1649**			**1609**			**1.49**	**[0.48; 2.50]**	**100.0%**

Heterogeneity: $I^2 = 98\%$, $\tau^2 = 6.2913$, $p < 0.01$
Test for overall effect: $z = 2.89$ ($p < 0.01$)
Test for subgroup differences: $\chi^2_2 = 0.08$, df = 2 ($p = 0.96$)

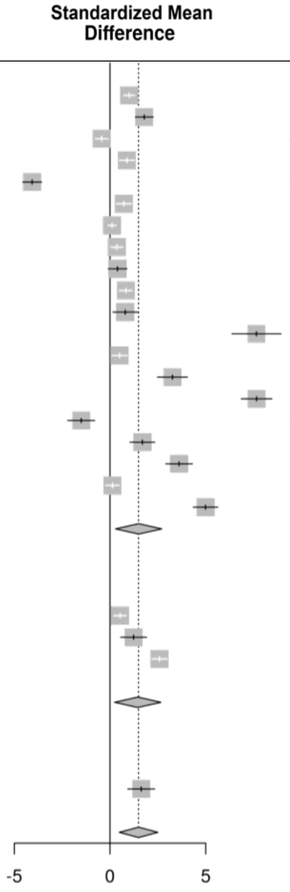

B

Study	PLWH ART Total	Mean	SD	HIV−negative Total	Mean	SD	SMD	95%-CI	Weight
subgroup = Greater than 100									
Abdulmumin 2024	100	31.60	13.5600	100	21.30	5.3000	1.00	[0.71; 1.29]	4.2%
Deshmukh 2024	150	28.94	7.4770	50	25.02	7.5230	0.52	[0.20; 0.85]	4.2%
Odegbemi 2024	120	21.78	7.3000	50	25.24	9.2700	-0.44	[-0.77; -0.10]	4.2%
Gbolahan 2023	59	36.91	25.0300	50	20.17	6.7000	0.88	[0.49; 1.28]	4.2%
Gospel 2023	100	12.83	0.4278	100	15.40	0.7841	-4.07	[-4.56; -3.58]	4.2%
Mutuma 2023	47	29.00	7.2750	51	23.90	6.9500	0.72	[0.31; 1.13]	4.2%
Tamuno-Boma 2023	165	7.45	3.4223	165	7.10	3.3675	0.10	[-0.11; 0.32]	4.2%
Younis 2022	101	23.00	14.0000	70	19.00	6.0000	0.35	[0.04; 0.66]	4.2%
Quaye 2019	105	39.51	16.9300	60	27.00	11.2000	0.83	[0.50; 1.16]	4.2%
Olisekodiaka 2018	40	29.60	2.5300	40	7.70	3.1600	7.65	[6.37; 8.93]	4.0%
Ashakiran 2018	90	46.90	6.6800	90	28.30	7.6800	2.58	[2.19; 2.98]	4.2%
Emokpae 2018	50	22.26	3.2200	50	21.08	0.9600	0.50	[0.10; 0.89]	4.2%
Ebot 2015	100	38.74	2.3900	100	23.98	1.3100	7.66	[6.85; 8.46]	4.1%
Ayelagbe 2014	45	27.40	6.3000	40	9.90	2.3000	3.61	[2.91; 4.30]	4.1%
Ibeh 2013	50	34.31	52.2100	100	25.00	80.0000	0.13	[-0.21; 0.47]	4.2%
Analike 2008	57	22.30	4.7000	100	6.10	2.0000	4.99	[4.35; 5.64]	4.2%
Random effects model	1379			1216			1.66	[0.20; 3.13]	66.8%
Heterogeneity: $I^2 = 99\%$, $\tau^2 = 8.8139$, $p < 0.01$									
subgroup = Less than 100									
Peter 2024	52	59.26	32.6200	50	13.82	14.5400	1.79	[1.33; 2.25]	4.2%
Ambad 2021	20	58.55	36.6100	20	24.31	14.2000	1.23	[0.55; 1.91]	4.1%
Ezeugwunne 2021	45	9.34	6.6400	26	6.53	8.0300	0.39	[-0.10; 0.88]	4.2%
Agbecha 2018	20	27.80	6.5000	20	23.46	4.2100	0.79	[0.15; 1.44]	4.2%
Aniagolu 2017	43	25.60	4.2000	20	13.95	1.4000	3.26	[2.48; 4.05]	4.1%
Nwosu 2015	20	7.80	4.4000	20	20.90	11.5000	-1.50	[-2.21; -0.80]	4.1%
Abubakar 2014	25	9.86	1.0500	25	7.57	1.6000	1.69	[1.04; 2.34]	4.2%
Beitune 2007	45	28.93	2.2200	12	24.62	3.8900	1.63	[0.92; 2.34]	4.1%
Random effects model	270			193			1.16	[0.22; 2.09]	33.2%
Heterogeneity: $I^2 = 93\%$, $\tau^2 = 1.7192$, $p < 0.01$									
**Random effects model**	**1649**			**1409**			**1.49**	**[0.48; 2.50]**	**100.0%**

Heterogeneity: $I^2 = 98\%$, $\tau^2 = 6.2913$, $p < 0.01$
Test for overall effect: $z = 2.89$ ($p < 0.01$)
Test for subgroup differences: $\chi^2_1 = 0.33$, df = 1 ($p = 0.57$)

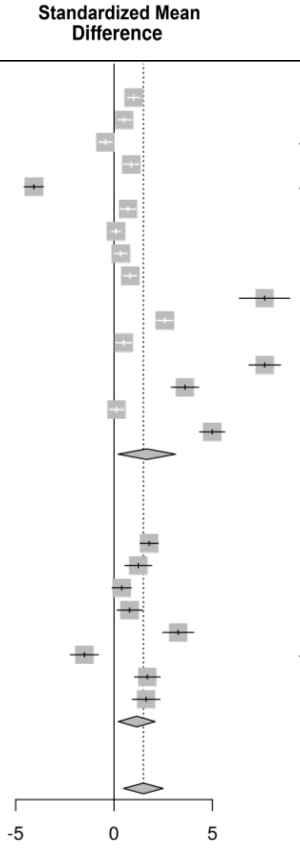

C

Supplementary File S2

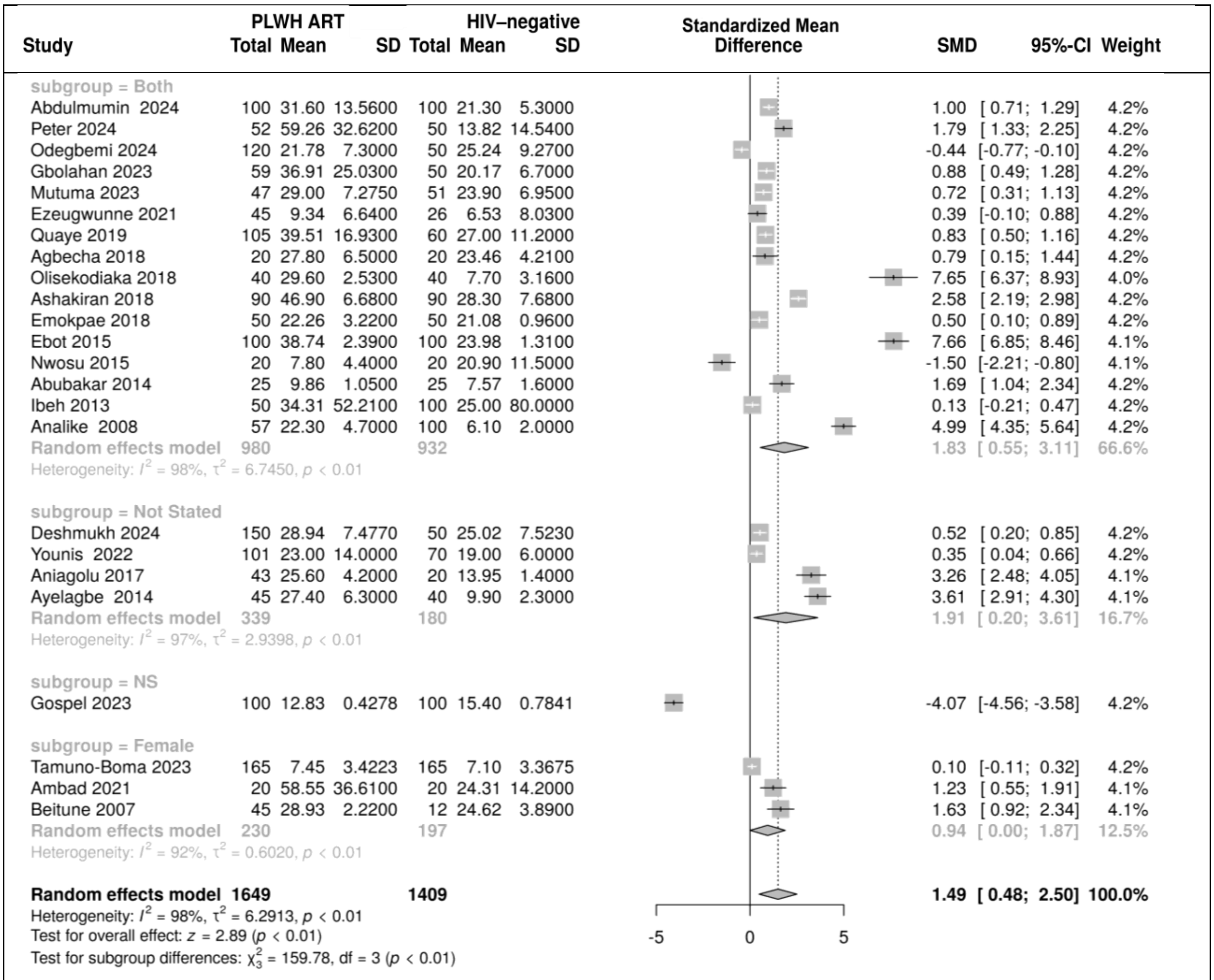

D

Supplementary File S2

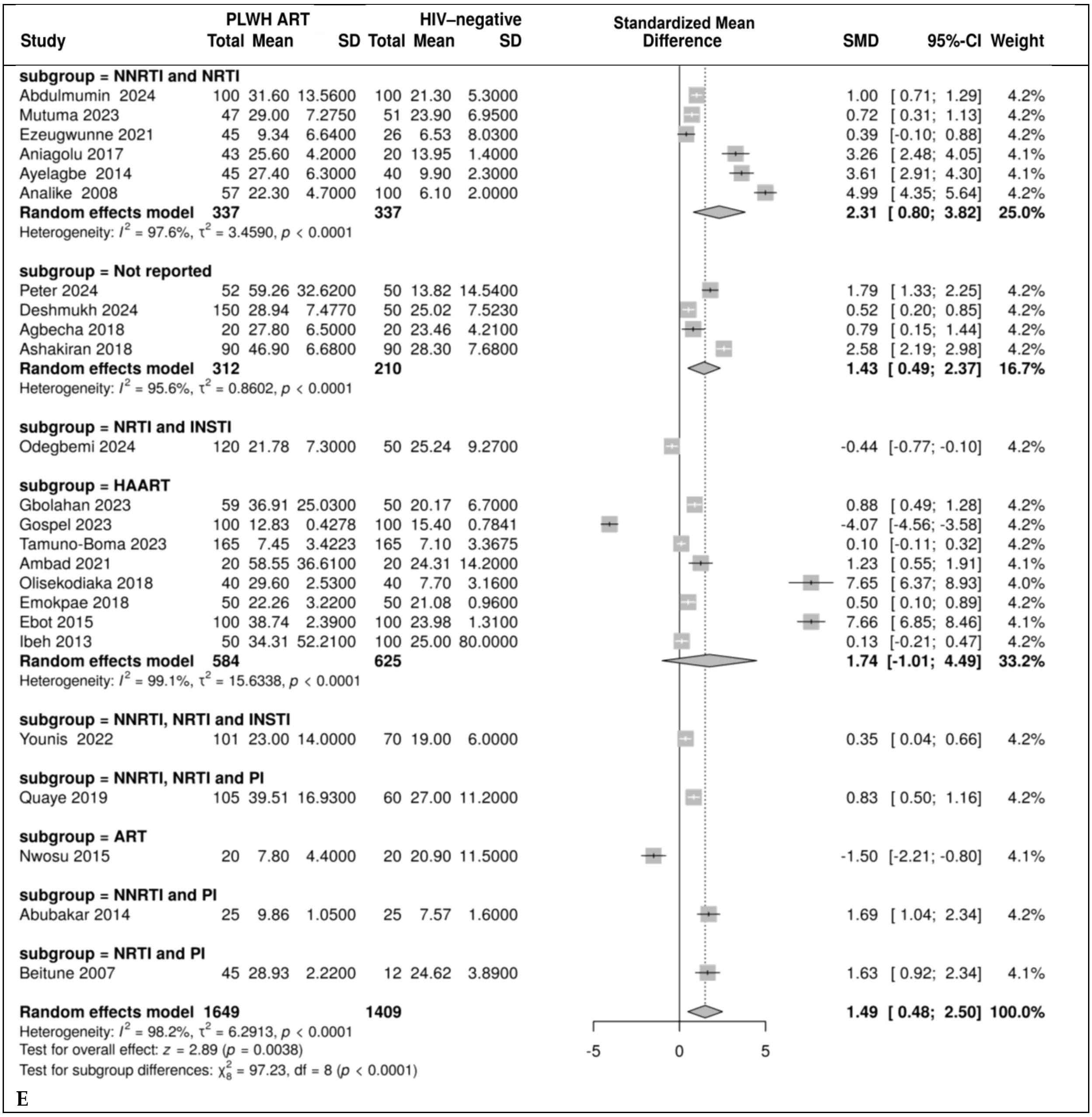

Figure S3: Subgroup analysis on AST in PLWH exposed to ART compared with HIV-negative individuals. A: AST in PLWH exposed to ART compared with HIV-negative individuals based on study design. B: AST in PLWH exposed to ART compared with HIV-negative individuals based on the continent of publication. C: AST in PLWH exposed to ART compared with HIV-negative individuals based on sample size. D: AST in PLWH exposed to ART compared with HIV-negative individuals based on gender distribution. E: AST in PLWH exposed to ART compared with HIV-negative individuals based on the class of ART. The solid line shows the line of no effect, the dashed line shows the effect size, the gray block shows the weight of the study, the horizontal line crossing the gray block shows the confidence intervals, diamond plot shows the combined effect size. NRTIs: nucleoside reverse transcriptase inhibitors, NNRTIs: non-nucleoside reverse transcriptase inhibitors, NtRTI: nucleotide reverse transcriptase inhibitors, PIs: protease inhibitors, INSTIs: integrase-nucleoside strand transfer inhibitors.

Supplementary File S2

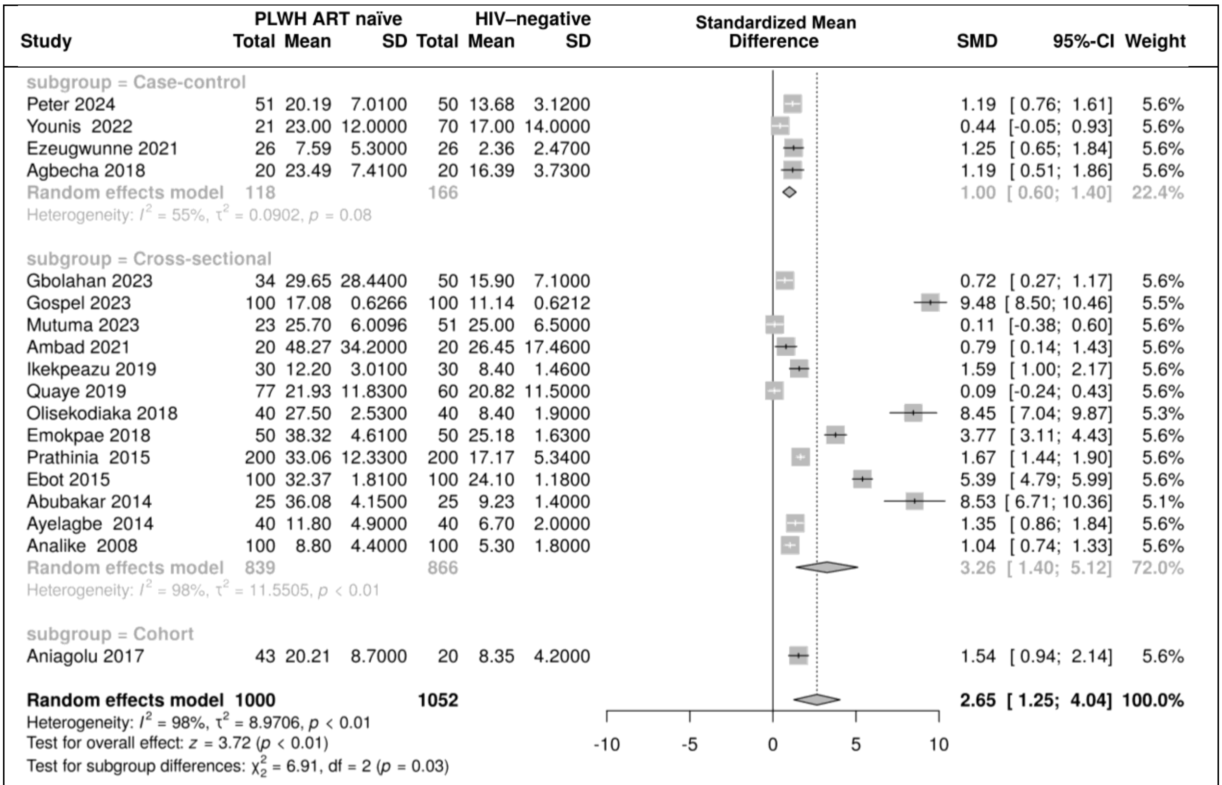

A

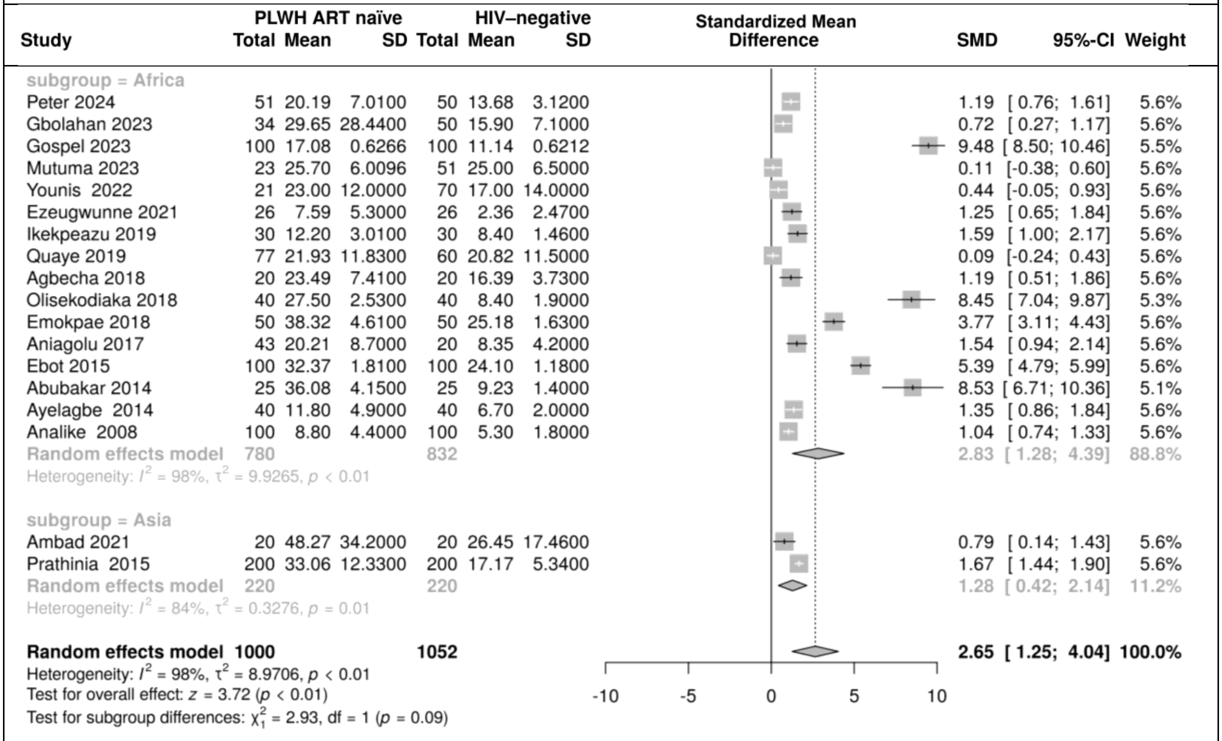

B

Supplementary File S2

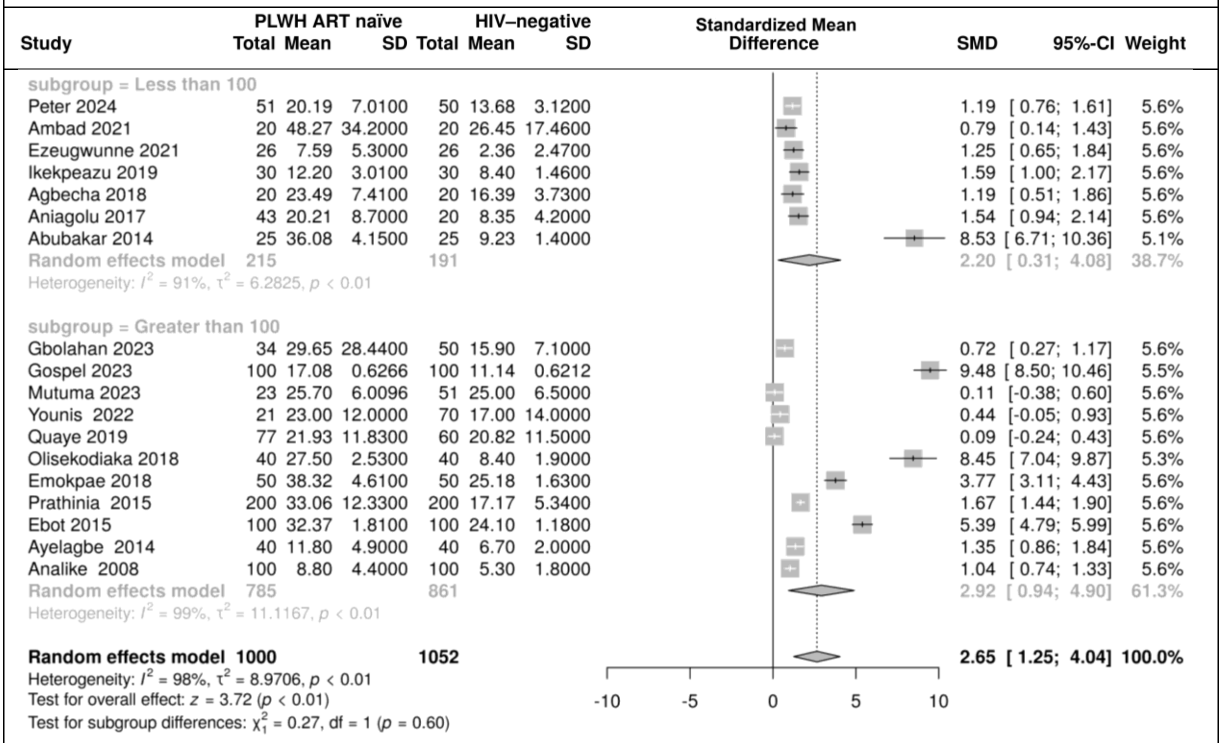

C

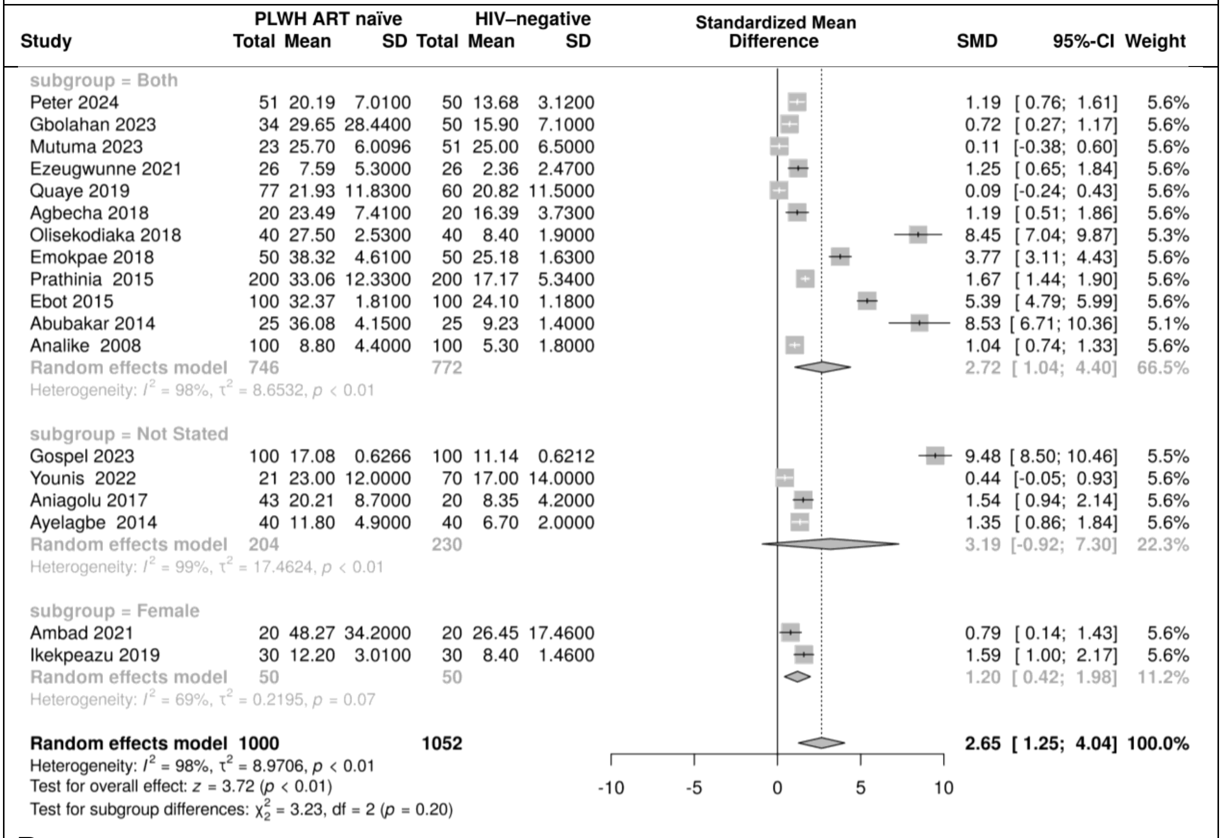

D

Supplementary File S2

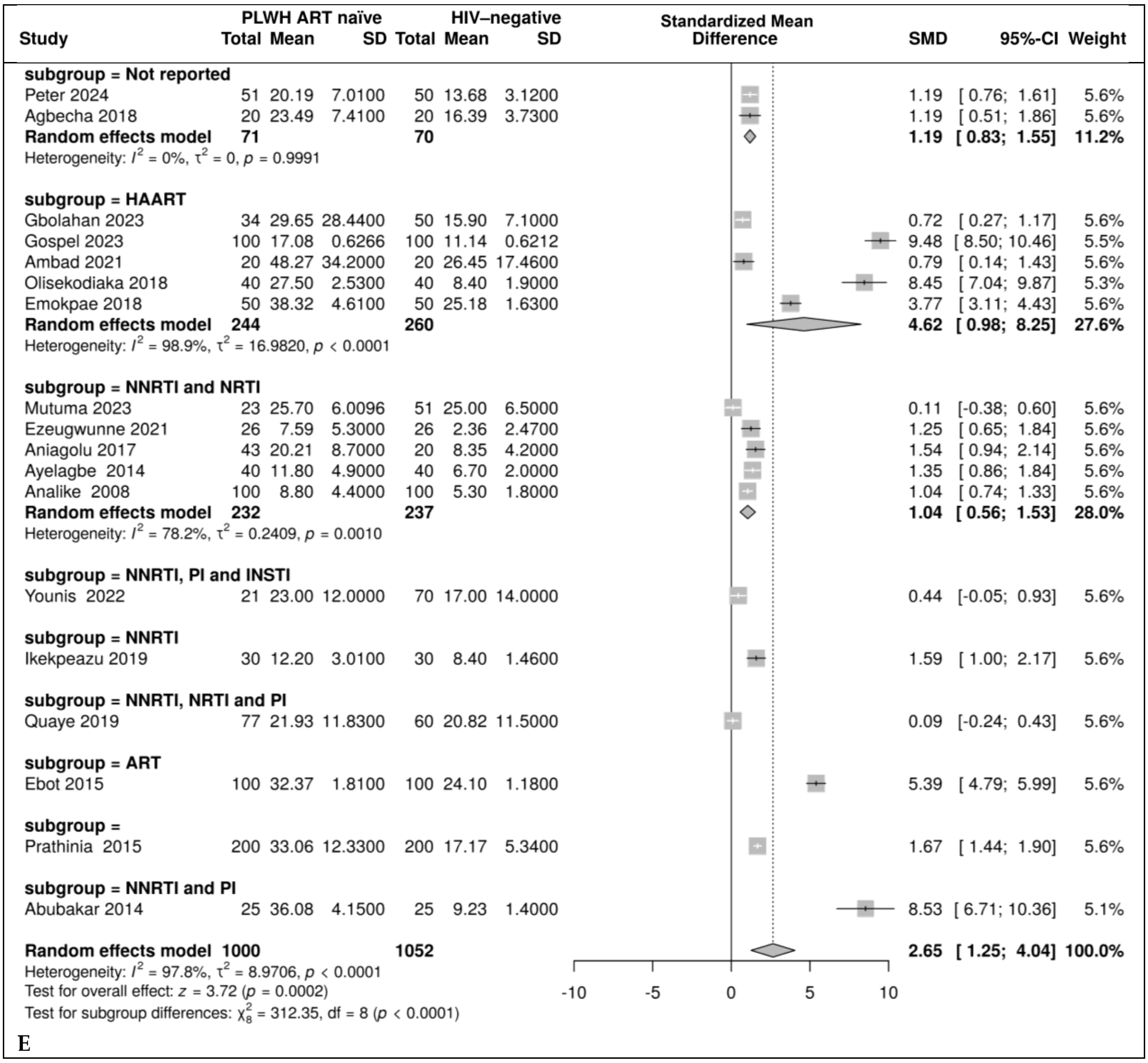

Figure S4: Subgroup analysis on ALT in ART-naïve compared with HIV-negative individuals. A: ALT level in PLWH on ART-naïve group compared with HIV negative, based on study design. B: Levels of ALT in ART-naïve PLWH compared with HIV negative, based on the continent of publication. C: ALT level in PLWH on ART-naïve compared with HIV negative, based on sample size. D: ALT levels in PLWH on ART-naïve compared with HIV negative, based on gender distribution. E: ALT levels in PLWH on ART-naïve group compared with HIV negative based on class of ART regimens. The solid line shows the line of no effect, the dashed line shows the effect size, the gray block shows the weight of the study, the horizontal line crossing the gray block shows the confidence intervals, diamond plot shows the combined effect size. NRTIs: nucleoside reverse transcriptase inhibitors, NNRTIs: non-nucleoside reverse transcriptase inhibitors, NtRTI: nucleotide reverse transcriptase inhibitors, PIs: protease inhibitors, INSTIs: integrase-nucleoside strand transfer inhibitors.

Supplementary File S2

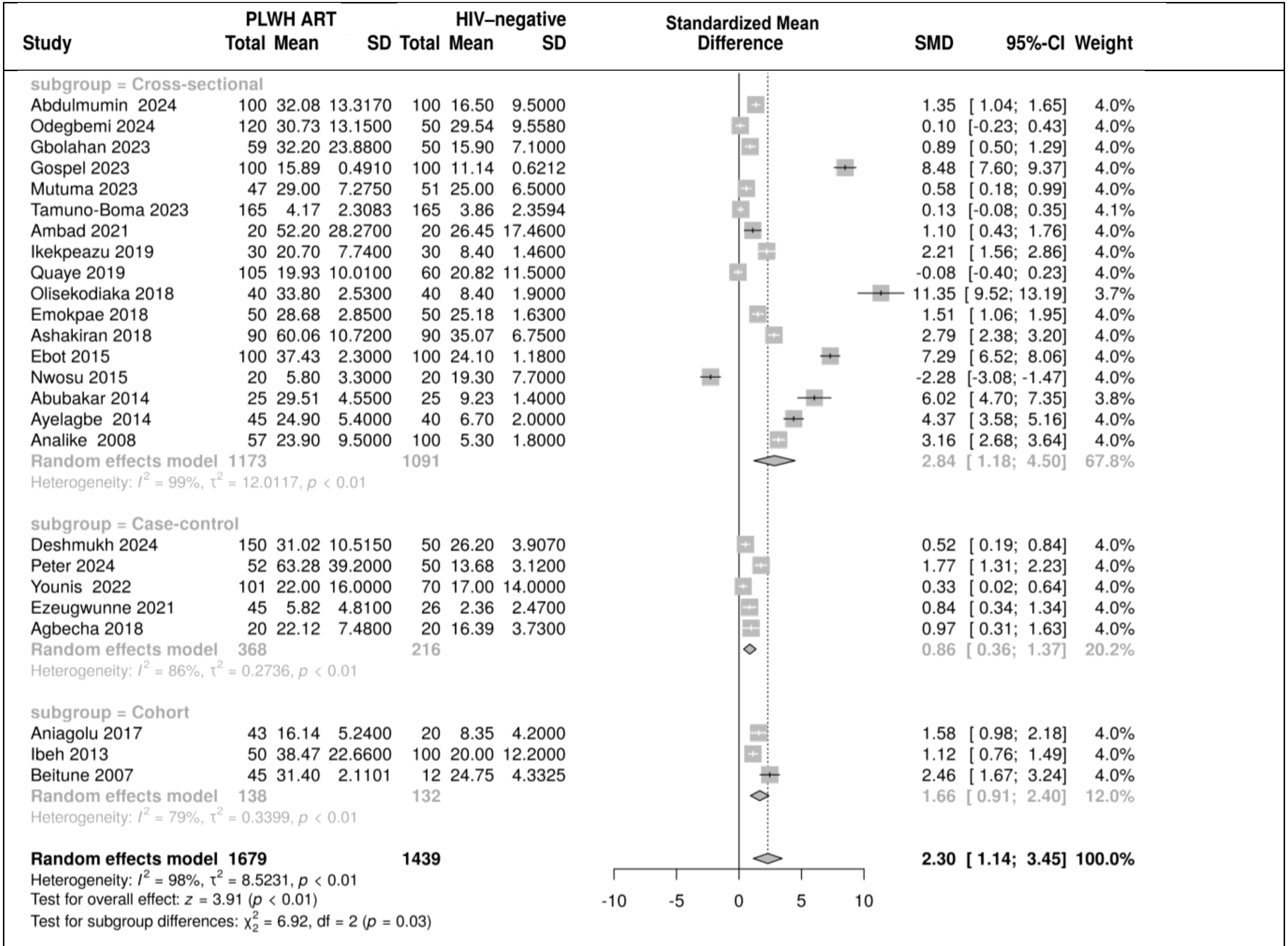

A

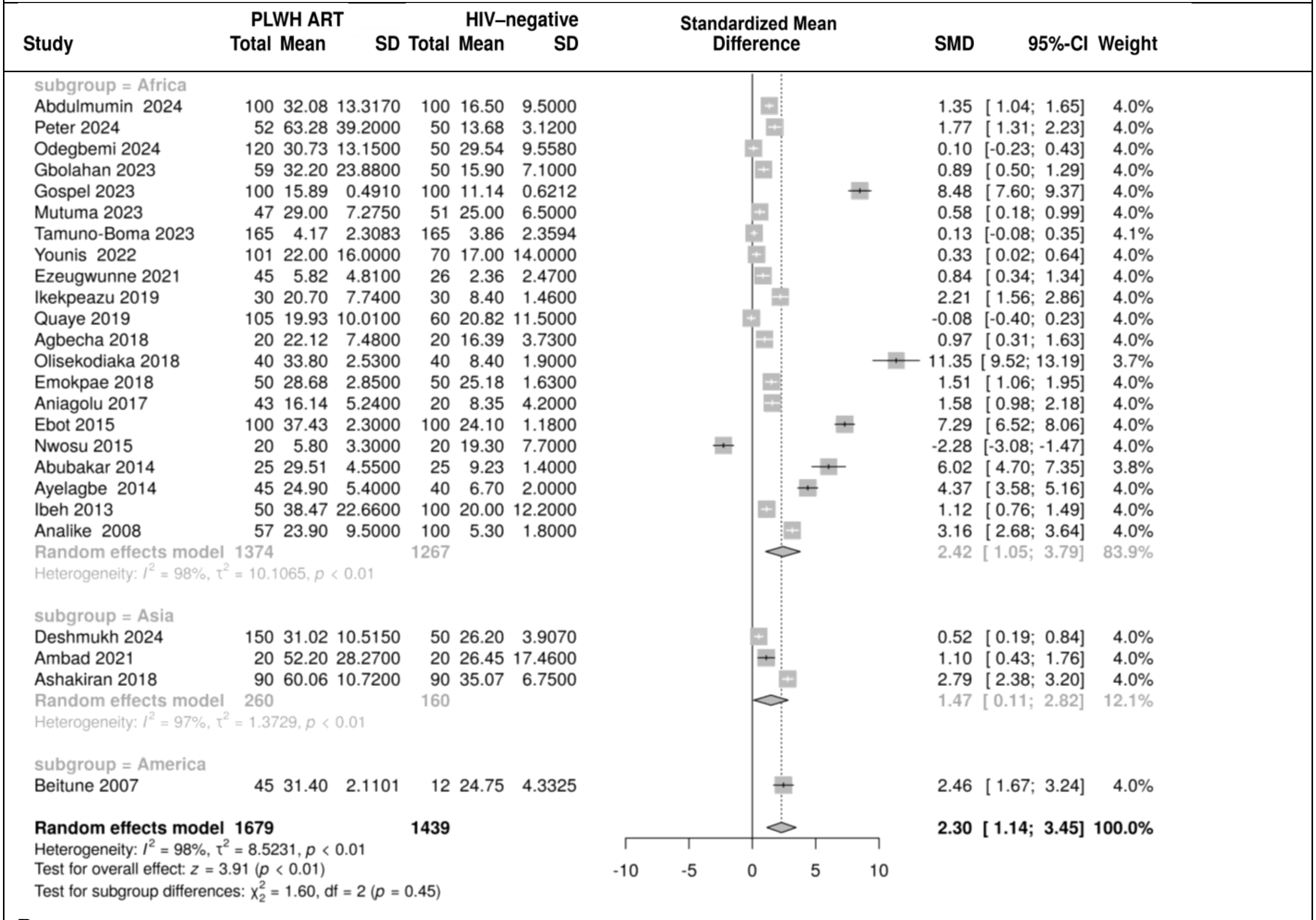

B

Supplementary File S2

Study	PLWH ART Total	PLWH ART Mean	PLWH ART SD	HIV−negative Total	HIV−negative Mean	HIV−negative SD	SMD	95%-CI	Weight
subgroup = Greater than 100									
Abdulmumin 2024	100	32.08	13.3170	100	16.50	9.5000	1.35	[1.04; 1.65]	4.0%
Deshmukh 2024	150	31.02	10.5150	50	26.20	3.9070	0.52	[0.19; 0.84]	4.0%
Odegbemi 2024	120	30.73	13.1500	50	29.54	9.5580	0.10	[-0.23; 0.43]	4.0%
Gbolahan 2023	59	32.20	23.8800	50	15.90	7.1000	0.89	[0.50; 1.29]	4.0%
Gospel 2023	100	15.89	0.4910	100	11.14	0.6212	8.48	[7.60; 9.37]	4.0%
Mutuma 2023	47	29.00	7.2750	51	25.00	6.5000	0.58	[0.18; 0.99]	4.0%
Tamuno-Boma 2023	165	4.17	2.3083	165	3.86	2.3594	0.13	[-0.08; 0.35]	4.1%
Younis 2022	101	22.00	16.0000	70	17.00	14.0000	0.33	[0.02; 0.64]	4.0%
Quaye 2019	105	19.93	10.0100	60	20.82	11.5000	-0.08	[-0.40; 0.23]	4.0%
Olisekodiaka 2018	40	33.80	2.5300	40	8.40	1.9000	11.35	[9.52; 13.19]	3.7%
Emokpae 2018	50	28.68	2.8500	50	25.18	1.6300	1.51	[1.06; 1.95]	4.0%
Ashakiran 2018	90	60.06	10.7200	90	35.07	6.7500	2.79	[2.38; 3.20]	4.0%
Ebot 2015	100	37.43	2.3000	100	24.10	1.1800	7.29	[6.52; 8.06]	4.0%
Ayelagbe 2014	45	24.90	5.4000	40	6.70	2.0000	4.37	[3.58; 5.16]	4.0%
Ibeh 2013	50	38.47	22.6600	100	20.00	12.2000	1.12	[0.76; 1.49]	4.0%
Analike 2008	57	23.90	9.5000	100	5.30	1.8000	3.16	[2.68; 3.64]	4.0%
Random effects model	1379			1216			2.70	[1.06; 4.34]	64.1%
Heterogeneity: $I^2 = 99\%$, $\tau^2 = 11.0931$, $p < 0.01$									
subgroup = Less than 100									
Peter 2024	52	63.28	39.2000	50	13.68	3.1200	1.77	[1.31; 2.23]	4.0%
Ambad 2021	20	52.20	28.2700	20	26.45	17.4600	1.10	[0.43; 1.76]	4.0%
Ezeugwunne 2021	45	5.82	4.8100	26	2.36	2.4700	0.84	[0.34; 1.34]	4.0%
Ikekpeazu 2019	30	20.70	7.7400	30	8.40	1.4600	2.21	[1.56; 2.86]	4.0%
Agbecha 2018	20	22.12	7.4800	20	16.39	3.7300	0.97	[0.31; 1.63]	4.0%
Aniagolu 2017	43	16.14	5.2400	20	8.35	4.2000	1.58	[0.98; 2.18]	4.0%
Nwosu 2015	20	5.80	3.3000	20	19.30	7.7000	-2.28	[-3.08; -1.47]	4.0%
Abubakar 2014	25	29.51	4.5500	25	9.23	1.4000	6.02	[4.70; 7.35]	3.8%
Beitune 2007	45	31.40	2.1101	12	24.75	4.3325	2.46	[1.67; 3.24]	4.0%
Random effects model	300			223			1.60	[0.24; 2.95]	35.9%
Heterogeneity: $I^2 = 95\%$, $\tau^2 = 4.1393$, $p < 0.01$									
**Random effects model**	**1679**			**1439**			**2.30**	**[1.14; 3.45]**	**100.0%**

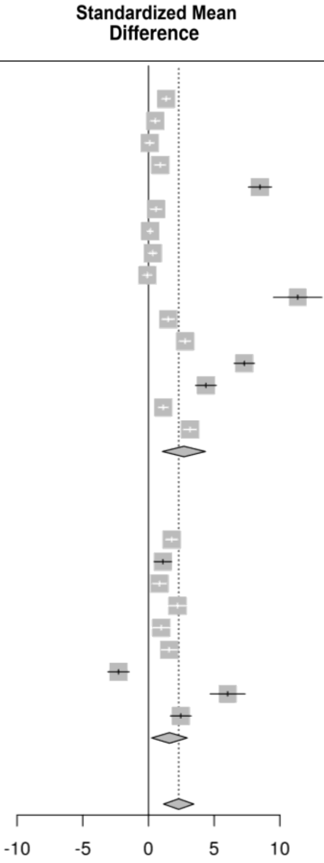

Heterogeneity: $I^2 = 98\%$, $\tau^2 = 8.5231$, $p < 0.01$
Test for overall effect: $z = 3.91$ ($p < 0.01$)
Test for subgroup differences: $\chi^2_1 = 1.03$, df = 1 ($p = 0.31$)

C

Supplementary File S2

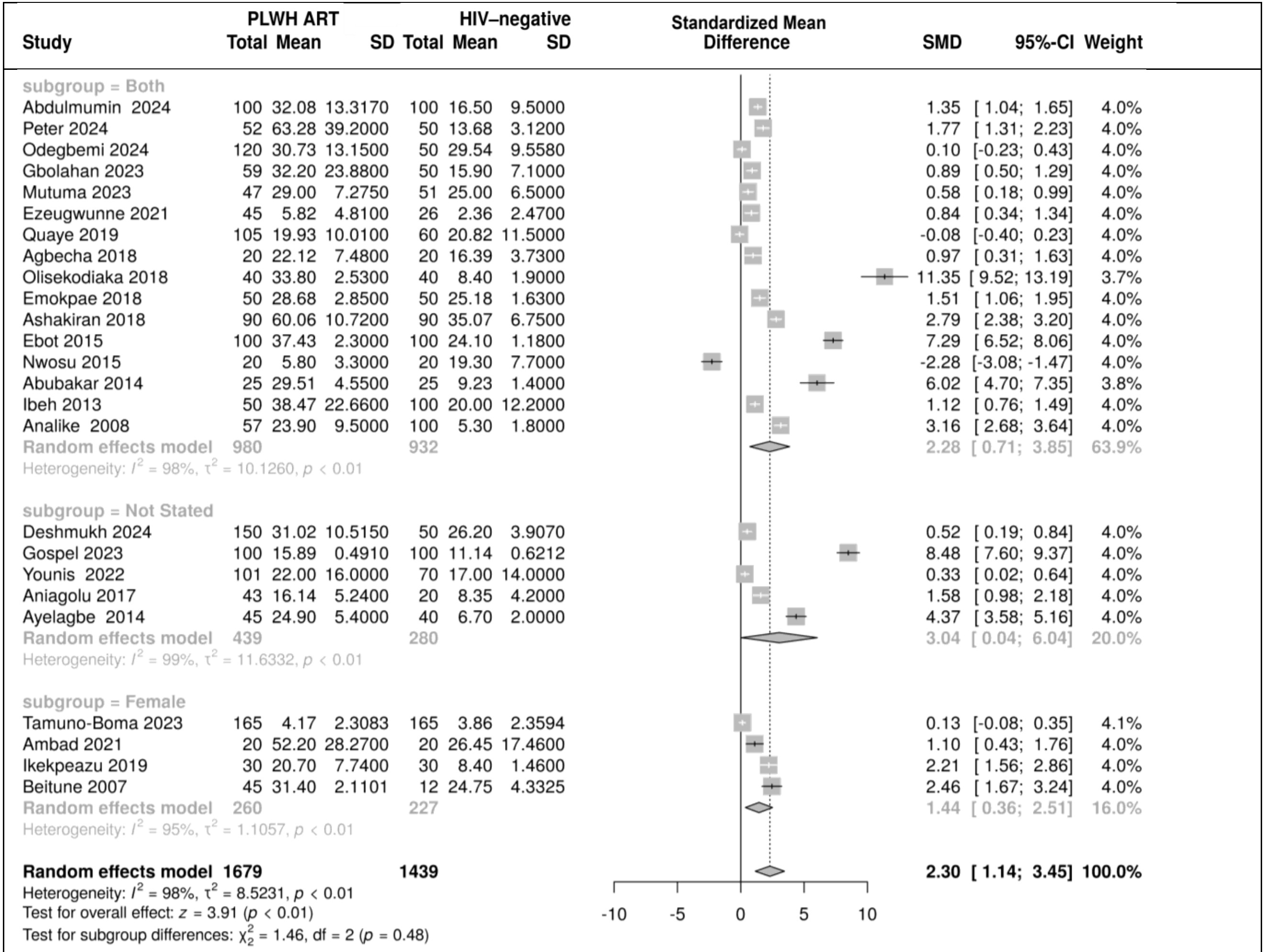

D

Supplementary File S2

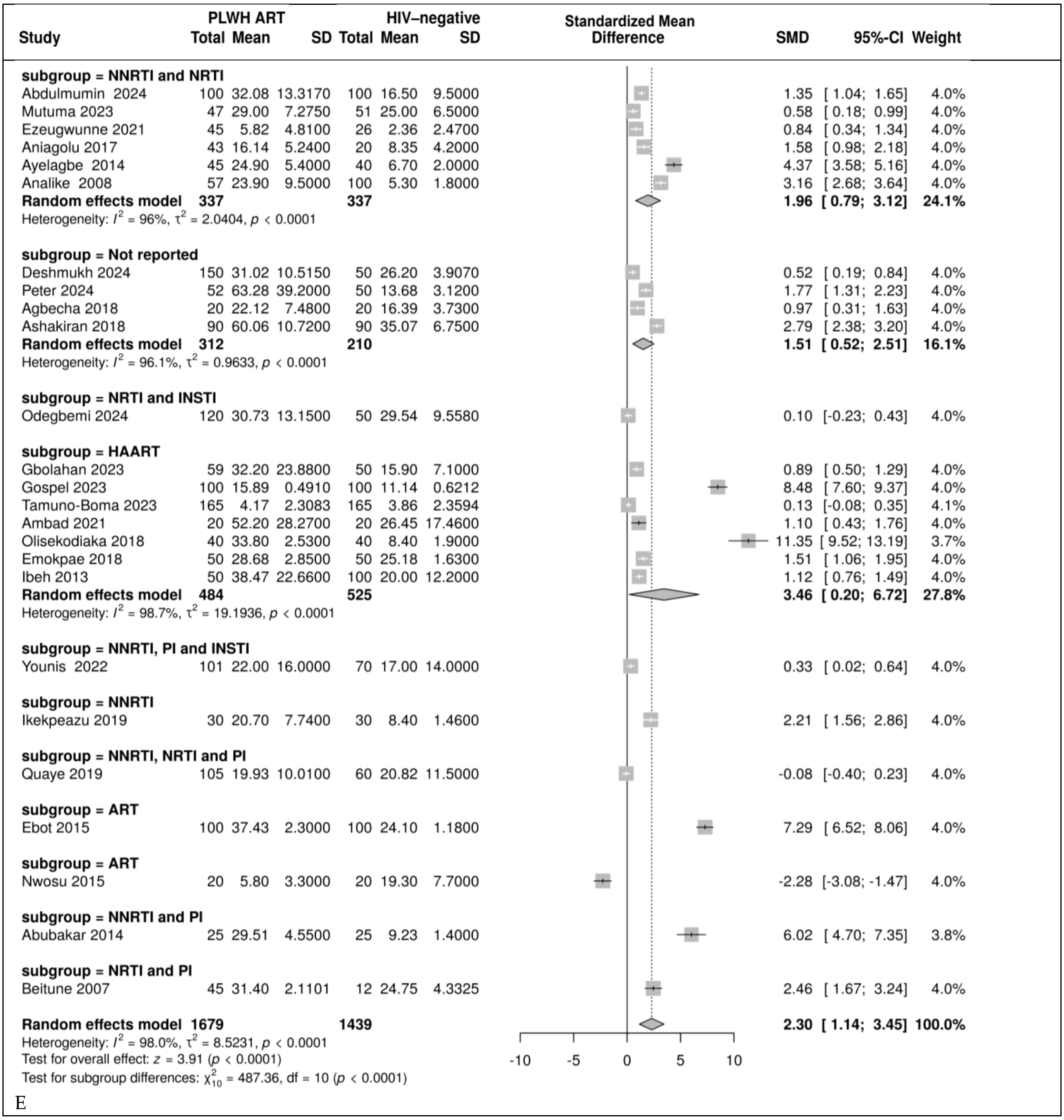

Figure S5: Subgroup analysis on ALT among PLWH on ART compared with HIV-negative individuals. A: ALT among PLWH on ART compared with HIV-negative individuals based on study design. B: ALT among PLWH on ART compared with HIV negative individuals based on continent. C: ALT among PLWH on ART compared with HIV-negative individuals based on sample size. D: ALT among PLWH on ART compared with HIV-negative individuals based on gender distribution. E: ALT among PLWH on ART compared with HIV-negative individuals based on the class of ART regimens. The solid line shows the line of no effect, the dashed line shows the effect size, the gray block shows the weight of the study, the horizontal line crossing the gray block shows the confidence intervals, diamond plot shows the combined effect size. NRTIs: nucleoside reverse transcriptase inhibitors, NNRTIs: non-nucleoside reverse transcriptase inhibitors, NtRTI: nucleotide reverse transcriptase inhibitors, PIs: protease inhibitors, INSTIs: integrase-nucleoside strand transfer inhibitors.

Supplementary File S2

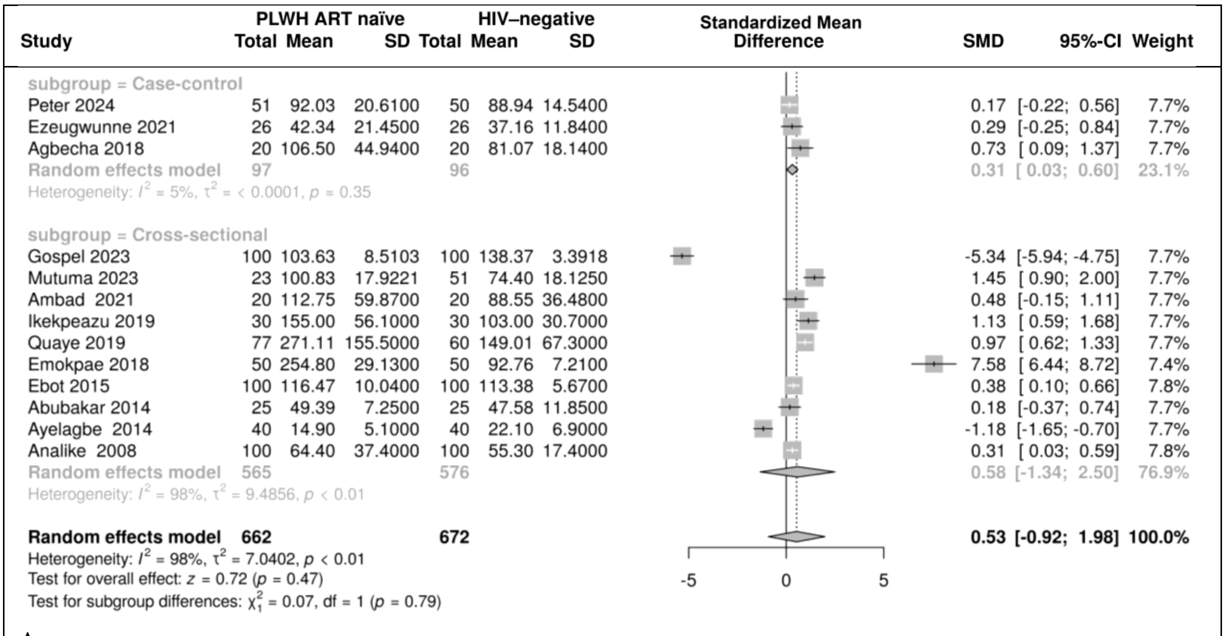

A

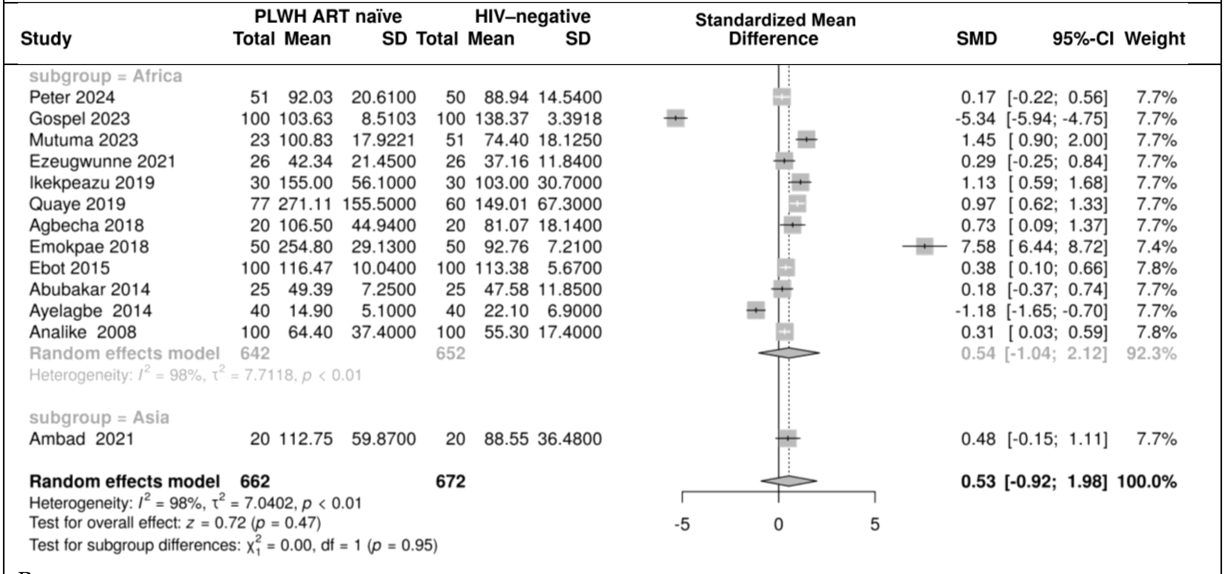

B

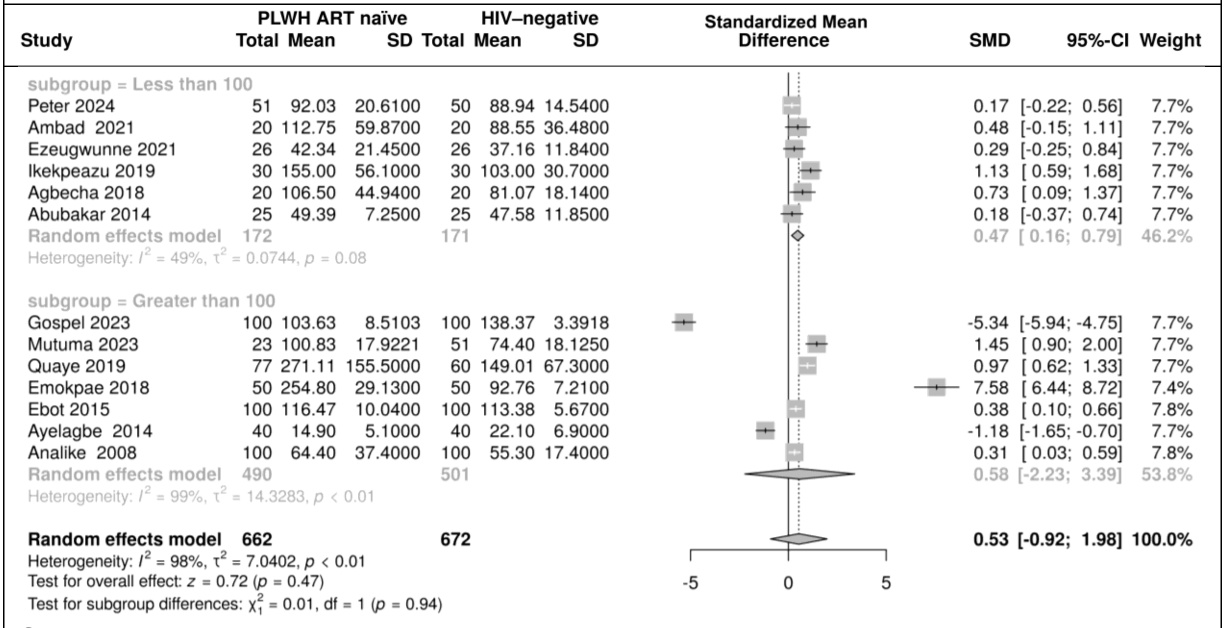

C

Supplementary File S2

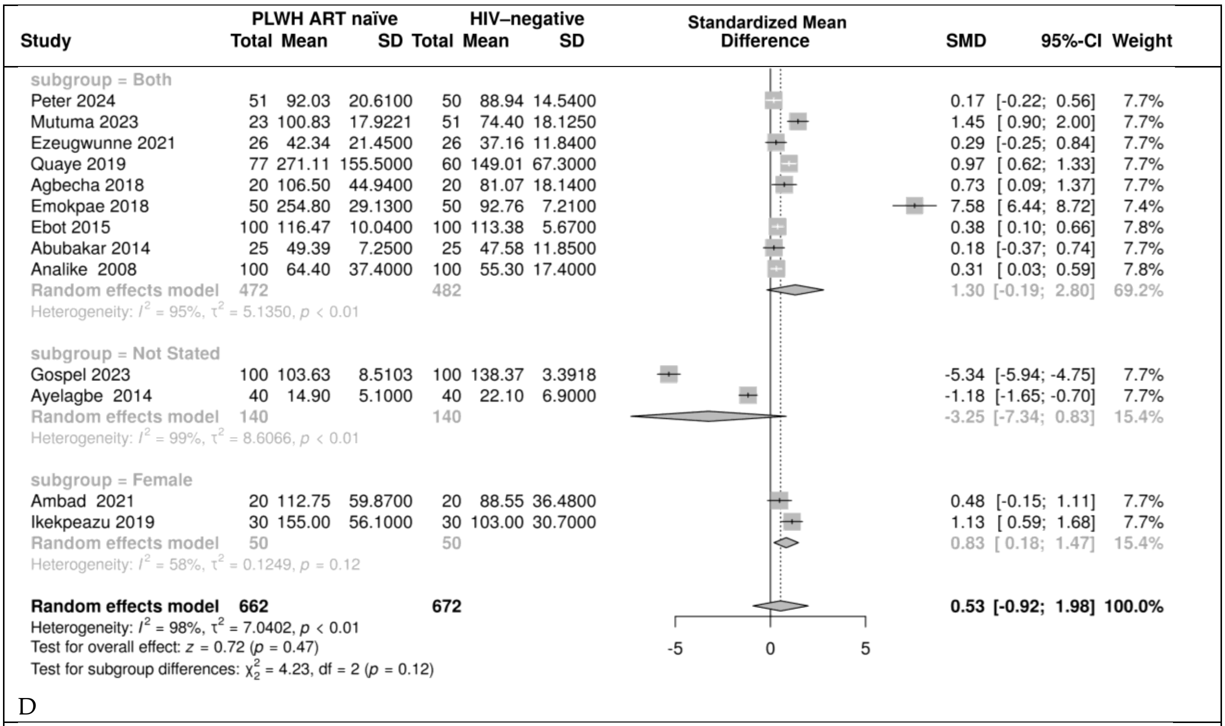

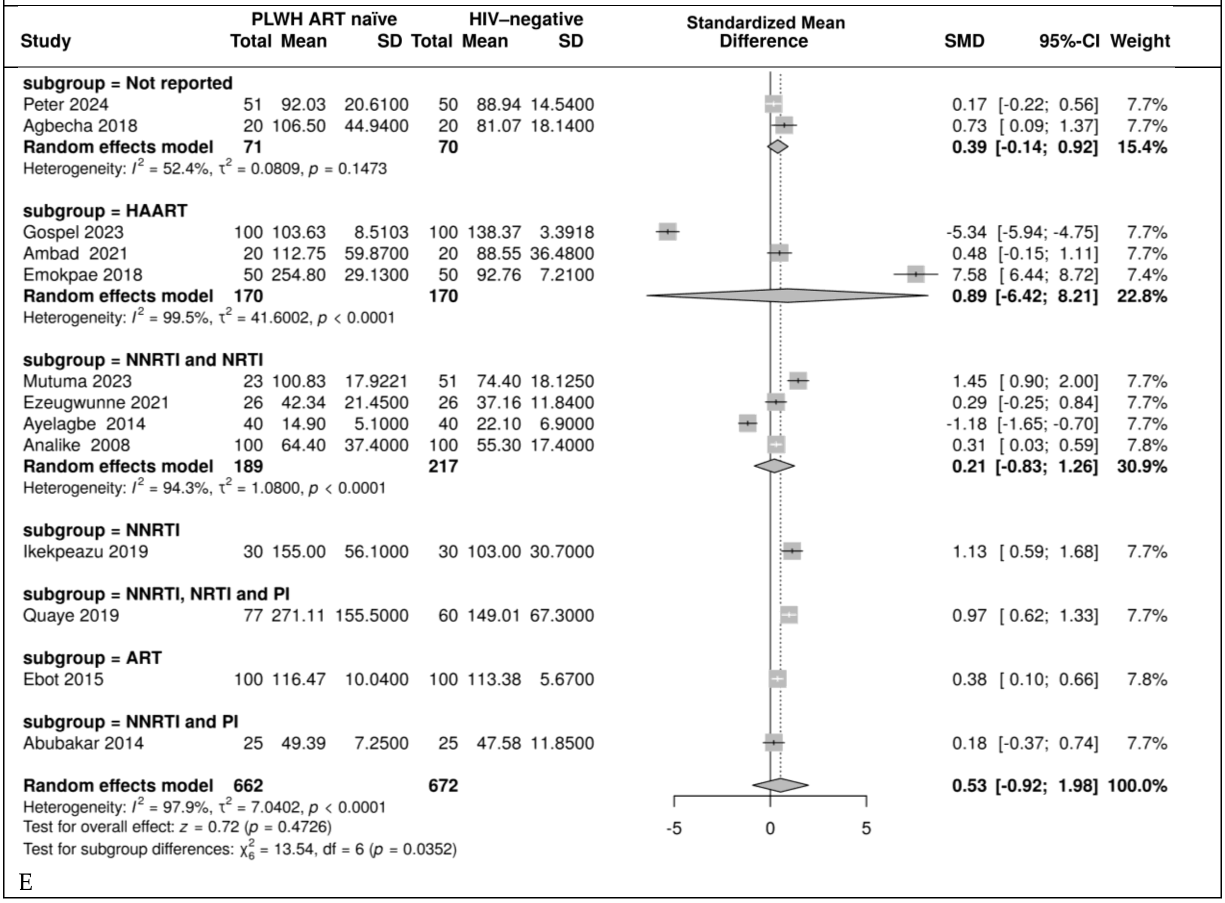

Figure S6: Subgroup analysis showing the effect of different factors on ALP in PLWH who are ART-naïve compared with HIV negative. A: ALP levels in PLWH who are ART-naïve compared with HIV-negative individuals, based on study design. B: ALP level in PLWH who are ART-naïve vs. HIV negative, based on the continent of publication. C: ALP levels in PLWH who are ART-naïve compared with HIV negative, based on sample size. D: ALP levels in PLWH who are ART-naïve compared with HIV negative, based on gender. E: ALP levels in PLWH who are ART-naïve compared with HIV negative, based on the class of ART regimens. The solid line shows the line of no effect, the dashed line shows the effect size, the gray block shows the weight of the study, the horizontal line crossing the gray block shows the confidence intervals, diamond plot shows the combined effect size. NRTIs: nucleoside reverse transcriptase inhibitors, NNRTIs: non-nucleoside reverse transcriptase inhibitors, NtRTI: nucleotide reverse transcriptase inhibitors, PIs: protease inhibitors, INSTIs: integrase-nucleoside strand transfer inhibitors.

Supplementary File S2

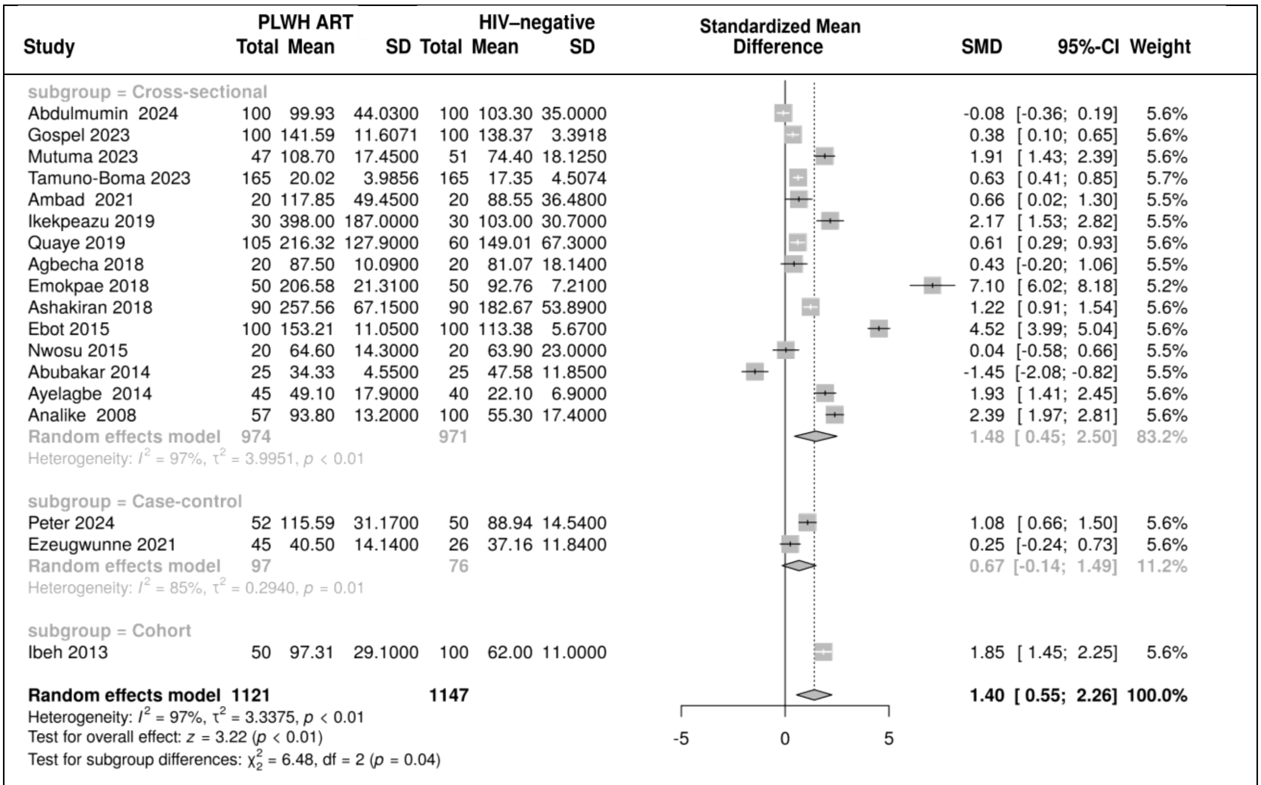

A

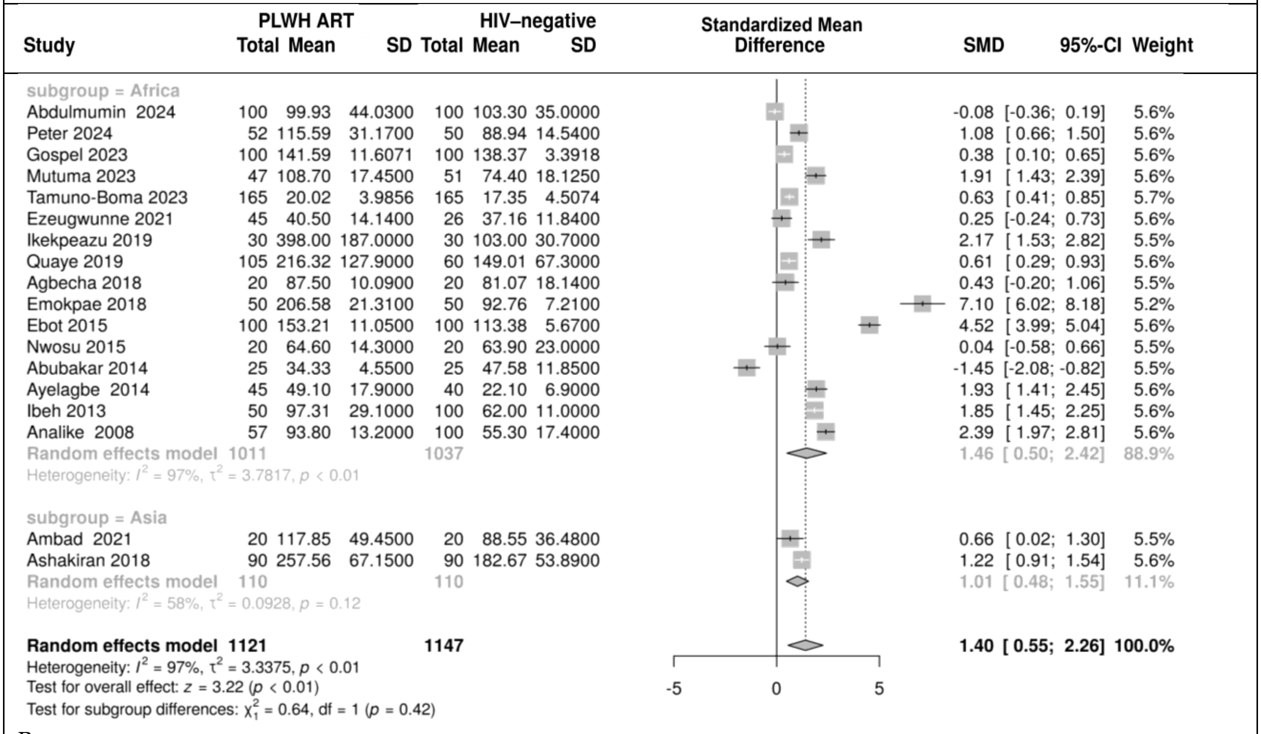

B

Supplementary File S2

Study	PLWH ART Total	PLWH ART Mean	PLWH ART SD	HIV–negative Total	HIV–negative Mean	HIV–negative SD	SMD	95%-CI	Weight
subgroup = Both									
Abdulmumin 2024	100	99.93	44.0300	100	103.30	35.0000	-0.08	[-0.36; 0.19]	5.6%
Peter 2024	52	115.59	31.1700	50	88.94	14.5400	1.08	[0.66; 1.50]	5.6%
Mutuma 2023	47	108.70	17.4500	51	74.40	18.1250	1.91	[1.43; 2.39]	5.6%
Ezeugwunne 2021	45	40.50	14.1400	26	37.16	11.8400	0.25	[-0.24; 0.73]	5.6%
Quaye 2019	105	216.32	127.9000	60	149.01	67.3000	0.61	[0.29; 0.93]	5.6%
Agbecha 2018	20	87.50	10.0900	20	81.07	18.1400	0.43	[-0.20; 1.06]	5.5%
Emokpae 2018	50	206.58	21.3100	50	92.76	7.2100	7.10	[6.02; 8.18]	5.2%
Ashakiran 2018	90	257.56	67.1500	90	182.67	53.8900	1.22	[0.91; 1.54]	5.6%
Ebot 2015	100	153.21	11.0500	100	113.38	5.6700	4.52	[3.99; 5.04]	5.6%
Nwosu 2015	20	64.60	14.3000	20	63.90	23.0000	0.04	[-0.58; 0.66]	5.5%
Abubakar 2014	25	34.33	4.5500	25	47.58	11.8500	-1.45	[-2.08; -0.82]	5.5%
Ibeh 2013	50	97.31	29.1000	100	62.00	11.0000	1.85	[1.45; 2.25]	5.6%
Analike 2008	57	93.80	13.2000	100	55.30	17.4000	2.39	[1.97; 2.81]	5.6%
Random effects model	761			792			1.51	[0.34; 2.68]	72.1%
Heterogeneity: $I^2 = 98\%$, $\tau^2 = 4.5785$, $p < 0.01$									
subgroup = Not Stated									
Gospel 2023	100	141.59	11.6071	100	138.37	3.3918	0.38	[0.10; 0.65]	5.6%
Ayelagbe 2014	45	49.10	17.9000	40	22.10	6.9000	1.93	[1.41; 2.45]	5.6%
Random effects model	145			140			1.14	[-0.39; 2.66]	11.2%
Heterogeneity: $I^2 = 96\%$, $\tau^2 = 1.1631$, $p < 0.01$									
subgroup = Female									
Tamuno-Boma 2023	165	20.02	3.9856	165	17.35	4.5074	0.63	[0.41; 0.85]	5.7%
Ambad 2021	20	117.85	49.4500	20	88.55	36.4800	0.66	[0.02; 1.30]	5.5%
Ikekpeazu 2019	30	398.00	187.0000	30	103.00	30.7000	2.17	[1.53; 2.82]	5.5%
Random effects model	215			215			1.13	[0.15; 2.11]	16.7%
Heterogeneity: $I^2 = 90\%$, $\tau^2 = 0.6750$, $p < 0.01$									
**Random effects model**	**1121**			**1147**			**1.40**	**[0.55; 2.26]**	**100.0%**

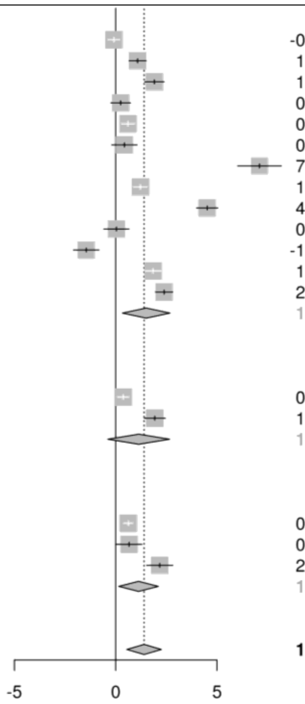

Heterogeneity: $I^2 = 97\%$, $\tau^2 = 3.3375$, $p < 0.01$
Test for overall effect: $z = 3.22$ ($p < 0.01$)
Test for subgroup differences: $\chi^2_2 = 0.27$, df = 2 ($p = 0.88$)

C

Study	PLWH ART Total	PLWH ART Mean	PLWH ART SD	HIV–negative Total	HIV–negative Mean	HIV–negative SD	SMD	95%-CI	Weight
subgroup = Greater than 100									
Abdulmumin 2024	100	99.93	44.0300	100	103.30	35.0000	-0.08	[-0.36; 0.19]	5.6%
Gospel 2023	100	141.59	11.6071	100	138.37	3.3918	0.38	[0.10; 0.65]	5.6%
Mutuma 2023	47	108.70	17.4500	51	74.40	18.1250	1.91	[1.43; 2.39]	5.6%
Tamuno-Boma 2023	165	20.02	3.9856	165	17.35	4.5074	0.63	[0.41; 0.85]	5.7%
Quaye 2019	105	216.32	127.9000	60	149.01	67.3000	0.61	[0.29; 0.93]	5.6%
Emokpae 2018	50	206.58	21.3100	50	92.76	7.2100	7.10	[6.02; 8.18]	5.2%
Ashakiran 2018	90	257.56	67.1500	90	182.67	53.8900	1.22	[0.91; 1.54]	5.6%
Ebot 2015	100	153.21	11.0500	100	113.38	5.6700	4.52	[3.99; 5.04]	5.6%
Ayelagbe 2014	45	49.10	17.9000	40	22.10	6.9000	1.93	[1.41; 2.45]	5.6%
Ibeh 2013	50	97.31	29.1000	100	62.00	11.0000	1.85	[1.45; 2.25]	5.6%
Analike 2008	57	93.80	13.2000	100	55.30	17.4000	2.39	[1.97; 2.81]	5.6%
Random effects model	909			956			2.01	[0.81; 3.21]	61.3%
Heterogeneity: $I^2 = 98\%$, $\tau^2 = 4.0769$, $p < 0.01$									
subgroup = Less than 100									
Peter 2024	52	115.59	31.1700	50	88.94	14.5400	1.08	[0.66; 1.50]	5.6%
Ambad 2021	20	117.85	49.4500	20	88.55	36.4800	0.66	[0.02; 1.30]	5.5%
Ezeugwunne 2021	45	40.50	14.1400	26	37.16	11.8400	0.25	[-0.24; 0.73]	5.6%
Ikekpeazu 2019	30	398.00	187.0000	30	103.00	30.7000	2.17	[1.53; 2.82]	5.5%
Agbecha 2018	20	87.50	10.0900	20	81.07	18.1400	0.43	[-0.20; 1.06]	5.5%
Nwosu 2015	20	64.60	14.3000	20	63.90	23.0000	0.04	[-0.58; 0.66]	5.5%
Abubakar 2014	25	34.33	4.5500	25	47.58	11.8500	-1.45	[-2.08; -0.82]	5.5%
Random effects model	212			191			0.46	[-0.35; 1.26]	38.7%
Heterogeneity: $I^2 = 92\%$, $\tau^2 = 1.0934$, $p < 0.01$									
**Random effects model**	**1121**			**1147**			**1.40**	**[0.55; 2.26]**	**100.0%**

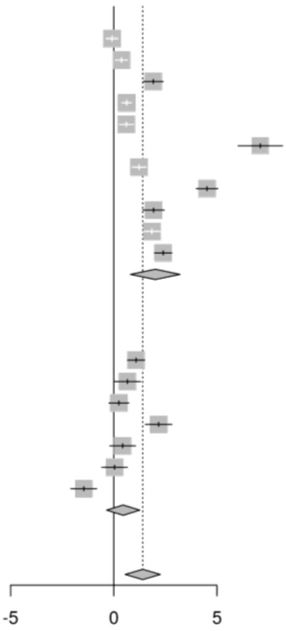

Heterogeneity: $I^2 = 97\%$, $\tau^2 = 3.3375$, $p < 0.01$
Test for overall effect: $z = 3.22$ ($p < 0.01$)
Test for subgroup differences: $\chi^2_1 = 4.42$, df = 1 ($p = 0.04$)

D

Supplementary File S2

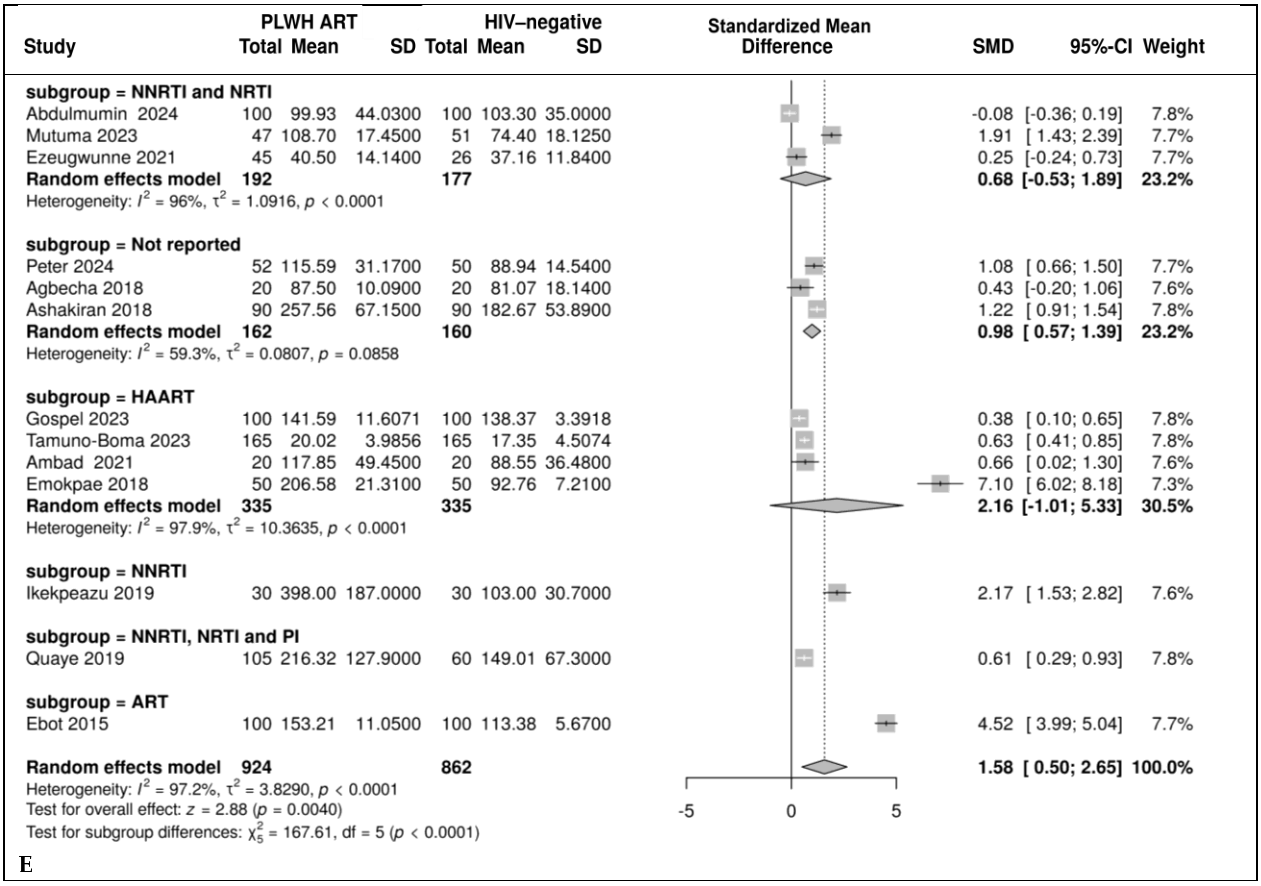

Figure S7: Subgroup analysis showing the effect of different factors on ALP in PLWH on ART compared with HIV negative. A: ALP levels in PLWH on ART compared with HIV-negative individuals, based on study design. B: ALP levels in PLWH on ART compared with HIV-negative individuals in terms of the continent of publication. C: ALP levels in PLWH on ART compared with HIV-negative individuals based on gender. D: ALP levels in PLWH on ART compared with HIV-negative individuals based on sample size. E: ALP levels in PLWH on ART compared with HIV-negative individuals based on class of ART regimens. The solid line shows the line of no effect, the dashed line shows the effect size, the gray block shows the weight of the study, the horizontal line crossing the gray block shows the confidence intervals, diamond plot shows the combined effect size. NRTIs: nucleoside reverse transcriptase inhibitors, NNRTIs: non-nucleoside reverse transcriptase inhibitors, NtRTI: nucleotide reverse transcriptase inhibitors, PIs: protease inhibitors, INSTIs: integrase-nucleoside strand transfer inhibitors.

Supplementary File S2

References

1. Abdulmumin, Y.; Haruna, I.U.; Danjaji, H.I.; Muhammad, M.; Mikail, T.A.; Rabiu, Z.; Lawan, U. Effect of Highly Active Antiretroviral Drugs Therapy (HAART) on Serum Hepatic and Renal Function Indices on HIV Patients in Kano Metropolitan. *Sahel Journal of Life Sciences FUDMA* 2024, *2*, 134–141, doi:10.33003/sajols-2024-0204-18.

2. Abriba, S.P.; Gambe, S.; Chindo, E.; Humphrey, Benedo.O. Evaluation of Some Liver Enzymes in HIV/AIDS Patients on Antiretroviral Therapy in University of Abuja Teaching Hospital, Nigeria. *International Journal of Human and Health Sciences (IJHHS)* 2024, *8*, 126–131, doi:10.31344/ijhhs.v8i2.632.

3. Deshmukh, H.; Patil, V.; Joshi, N.; Nagar, V. Relevance of Hepatic Enzymes in People Living with HIV on Antiretroviral Therapy. *Int. J Pharm Sci Rev Res* 2024, *84*, doi:10.47583/ijpsrr.2024.v84i02.015.

4. Odegbemi, O.B.; Olaniyan, M.F.; Muhibi, M.A. Hepatic Toxicity Assessment in HIV's Interaction with Reverse Transcriptase and Integrase Strand Transfer Inhibitors at a Military Hospital, Southsouth Nigeria. *Egyptian Liver Journal* 2024, *14*, 77, doi:10.1186/s43066-024-00377-w.

5. Tamuno-Boma, O.; Azuonwu, O.; Opusunju Boma, H.; Tee Popnen, G.; Gabriel-Brisibe, C.U.; Ihua, N.; Akuru Udiomine, B.; Akram, M. Assessment on Liver Function Biomarkers in HIV Positive Pregnant and Non-Pregnant Women on Antiretroviral Therapy in Rivers State, Nigeria. *J HIV Clin Sci Res* 2023, *10*, 001–005, doi:10.17352/2455-3786.000035.

6. Gbolahan, I.A.; Victoria, M.; Ugbomoiko, D.O.; Gambo, E.D.; Ibrahim, M.A. Estimation of Serum Minerals, Total Protein and Liver Enzymes in HIV Patients Receiving Haart in Federal Medical Centre, Keffi, Nasarawa State, Nigeria. *Asian Journal of Research in Biochemistry* 2023, *13*, 1–11, doi:10.9734/ajrb/2023/v13i3255.

7. Gospel, A.; Chimezie, D.N.; Chimerenka, J.I.; Tochukwu Nnadiukwu Effects of Anti-Retroviral Therapy on Some Liver Parameters of Hiv Sero-Positive Individuals in Rivers State, Nigeria. *International Journal of Advanced Academic Research |* 2023, *9*, 73–85.

8. Mutuma, B.; Omedo, R.; Wafula, P.; Demba, N.; Zablon, J.; Shaviya, N.; Were, T. Hepatic Function and Its Association with Clinical Outcomes in Non-Adherent HIV-1 Adults . *Afro-Egyptian Journal of Infectious and Endemic Diseases* 2023, *0*, 0–0, doi:10.21608/aeji.2023.188741.1288.

9. Younis, M.Y.G.; El-Sherif, M.; Alhaddad, A.B. Lipid Abnormalities among Libyan HIV-Infected Patients Receiving Antiretroviral (ARV) Drugs and ARV Naïve Patients. *J Adv Med Med Res* 2022, 470–481, doi:10.9734/jammr/2022/v34i234884.

10. Ezeugwunne, J.; Ogbodo, E.; Ezeuduji, O.; Iwuji, J.; Okwara, N.; Obi-Ezeani, C.; Amah, A.; Odumodu, I.; Izuchukwu, E. Assessment of Alpha-Fetoprotein, Albumin, Cd4+ and Some Liver Enzymes in HIV Infected Adult on Art in Nauth Nnewi, South Eastern Nigeria. 2021, *12*, 199–205.

11. Ambad, R.S.; Kumar Jha, R.; Bhatt, N.; Kumar Jha, R. Study on Activity of Liver Enzymes in HIV Affected Women. *Annals of R.S.C.B* 2021, *25*, 7093–7098.

12. Ikekpeazu, Joy.E.; Ibegbu, Madu.D.; Onyekwelu, Kenechukwu.C.; Uche, Ozichukwuamaka.S. Liver-Enzyme-Activities-in-Hiv-Seropositive-Pregnant-Women-on-Highly-Active-Antiretroviral-Therapy-Haart. *Int J HIV AIDS Res* 2019, *2*, 7–10.

13. Quaye, O.; Kuleape, J.A.; Bonney, E.Y.; Puplampu, P.; Tagoe, E.A. Imbalance of Antioxidant Enzymes Activities and Trace Elements Levels in Ghanaian HIV-Infected Patients. *PLoS One* 2019, *14*, doi:10.1371/journal.pone.0220181.

14. Emokpae, M.A.; Akhimien, J.O. Abnormal Biomarkers of Liver Function in Human Immunodeficiency Virus Type 1 Infected Subjects without Hepatitis B or C Co-Infection and Their Association with Disease Severity. *Journal of Medical Discovery* 2018, *3*, doi:10.24262/jmd.3.1.17058.

15. Olisekodiaka, M.J.; Onuegbu, A.; Igbeneghu C; Garuba, W.O.; Amah, U.; Okwara, J.E. Measurement of CD 4 + Cells and Liver Functions in HIV Patients on Antiretroviral Therapy. *Annals of International Medical and Dental Research* 2018, *4*, PT01–PT05.

16. Ashakiran, N.; A.R Satyanarayana, V.; Ravikanth, M.; S Girish Kumar, P. Abnormalities of Liver Enzymes in HIV Positive Patients on Antiretroviral Therapy. *International Journal of Clinical Biochemistry and Research* 2019, *6*, 61–63, doi:10.18231/2394-6377.2019.0016.

17. Agbecha, A.; Ikyernum, J. Impact of HIV-Infection on Serum Liver Enzymes: A Comparative Study among Anti-Retroviral Therapy (ART) Naïve Patients, ART Follow-Up Patients, and HIV Sero-Negative Controls. *Int J Healthc Med Sci* 2018, 196–200, doi:10.32861/ijhms.412.196.200.

18. Aniagolu, M.; Ugwuene, F.O.; Ikegwuonu, I. The Effects of Highly Active Antiretroviral Therapy on the Activities of Some Liver Enzymes and the Concentrations of Protein and Albumin in HIV Positive Patients in Nsukka South East Nigeria. *International Journal of Health Sciences & Research (www.ijhsr.org)* 2017, *7*, 67–71.

19. Ebot, W.; Achidi, E.; Kamga, H.-L.; Njunda, A.; Apinjoh, T. Liver Function Tests of HIV/AIDS Patients at the Nylon District Hospital, Douala, Cameroon. *Int J Res Med Sci* 2015, 2549–2552, doi:10.18203/2320-6012.ijrms20150788.

20. Prathinia, M.B.; Reshma, S.; Madan Gopal, R.; Sushith; Pravira, K.; Suriyan Nair Significance of Liver Enzymes as a Baseline Investigation in Recently Diagnosed HIV Positive Patients. *International Journal of Biomedical and Advance Research* 2015, *6*, 768–770.

21. Nwosu, D.C.; Okolie, N.J.C.; Ajero, C.M.U.; Ojiegbe, G.C.; Oze, G.O.; Ifeanyi, E.; Nnatunanya, I.; Amajuoyi, O.; Ochei, K.C.; Okpara, K.E. Biochemical Alteration in Adults HIV Patients on Antiretroviral Therapy. *Word Journal of Pharmacy and Pharmaceutical Sciencess* 2015, *4*, 153–160.

22. Ayelagbe, O.G.; Akerele, O.P.; Onuegbu, A.J.; Oparinde, D.P. Drug Hepatotoxicity in HIV Patients on Highly Active Antiretroviral Therapy [HAART] in Southwest Nigeria. *IOSR Journal of Dental and Medical Sciences* 2014, *13*, 67–70, doi:10.9790/0853-13566770.

23. Abubakar, M.; Abduljalil, M.; Nasiru, Y. Changes in Liver Function Enzymes of HIV/AIDS Patients Treated with Antiretroviral Drugs (ARVS) in Specialist Hospital. *Nigerian Journal of Basic and Applied Science* 2014, *22*, 85–89, doi:10.4314/njbas.v22i3.6.

24. Ibeh, B.O.; Omodamiro, O.D.; Ibeh, U.; Habu, J.B. Biochemical and Haematological Changes in HIV Subjects Receiving Winniecure Antiretroviral Drug in Nigeria. *J Biomed Sci* 2013, *20*, doi:10.1186/1423-0127-20-73.

25. Analike, R.; Nnamah, N.; Dioka, C.; Meludu, S.; Osuji, C.; Asomugha, A. Evaluation of Liver Function Tests of HIV Positive Patients on Antiretroviral Therapy in Nnewi,Nigeria. *Journal of Biomedical Investigation* 2008, *4*, 42–48, doi:10.4314/jbi.v4i2.30415.

26. El Beitune, P.; Duarte, G.; Campbell, O.; Quintana, S.M.; Rodrigues, L.C. *Effects of Antiretroviral Agents During Pregnancy on Liver Enzymes and Amylase in HIV-Exposed, Uninfected Newborn Infants*;.*Braz. J. Infect. Dis.* **2007**, *11*, 314–317.
